# Conotoxins: Classification, Prediction, and Future Directions in Bioinformatics

**DOI:** 10.3390/toxins17020078

**Published:** 2025-02-09

**Authors:** Rui Li, Junwen Yu, Dongxin Ye, Shanghua Liu, Hongqi Zhang, Hao Lin, Juan Feng, Kejun Deng

**Affiliations:** The Clinical Hospital of Chengdu Brain Science Institute, School of Life Science and Technology, Center for Informational Biology, University of Electronic Science and Technology of China, Chengdu 610054, China; 202321140412@std.uestc.edu.cn (R.L.); 202322140206@std.uestc.edu.cn (J.Y.); 202321140113@std.uestc.edu.cn (D.Y.); 202321140418@std.uestc.edu.cn (S.L.); 202321140413@std.uestc.edu.cn (H.Z.); hlin@uestc.edu.cn (H.L.); fengjuan@uestc.edu.cn (J.F.)

**Keywords:** conotoxins, venom peptide identification, computational peptide analysis, machine learning applications, peptide-based drug discovery

## Abstract

Conotoxins, a diverse family of disulfide-rich peptides derived from the venom of *Conus* species, have gained prominence in biomedical research due to their highly specific interactions with ion channels, receptors, and neurotransmitter systems. Their pharmacological properties make them valuable molecular tools and promising candidates for therapeutic development. However, traditional conotoxin classification and functional characterization remain labor-intensive, necessitating the increasing adoption of computational approaches. In particular, machine learning (ML) techniques have facilitated advancements in sequence-based classification, functional prediction, and de novo peptide design. This review explores recent progress in applying ML and deep learning (DL) to conotoxin research, comparing key databases, feature extraction techniques, and classification models. Additionally, we discuss future research directions, emphasizing the integration of multimodal data and the refinement of predictive frameworks to enhance therapeutic discovery.

## 1. Introduction

Conotoxins are small peptides found in the venom of cone snails (genus *Conus*), and they have attracted significant attention due to their potent pharmacological effects, particularly in modulating ion channels, receptors, and neurotransmitter systems [1]. They are important in regulating key biological processes, such as nerve signal transmission, making them valuable in fundamental and applied biomedical research. Given their high specificity and diverse mechanisms of action, conotoxins hold immense promise as therapeutic agents, particularly in pain management, neurological disorders, and other diseases with inadequate treatment options [2].

Despite their significant potential, the discovery, characterization, and therapeutic optimization of conotoxins face substantial challenges [3,4,5,6]. Traditionally, conotoxin discovery relies on extracting and isolating active components from *Conus* venom [7,8,9], followed by classification into various superfamilies and pharmacological families based on sequence homology and functional properties. However, traditional methods for analysis and identification are time-consuming and labor-intensive [10]. As a result, researchers have increasingly turned to computational approaches, particularly bioinformatics tools and machine learning (ML) techniques [11,12,13,14], to improve the classification, prediction, and generation efficiency of conotoxins [15,16,17,18,19,20,21,22,23,24,25,26,27].

Integrating large-scale databases, such as UniProt, ConoServer, and other specialized repositories, has significantly accelerated conotoxin research [28,29,30,31]. These resources provide comprehensive sequence, structure, and functional information, supporting the development of predictive models and facilitating the discovery of novel toxins. In recent years, machine learning and deep learning (DL) methods have proven highly effective [32,33,34,35], enabling researchers to predict the functions of conotoxins, identify potential receptor targets, and even generate new peptide sequences for drug development [36,37,38]. There remains considerable room for expanding the data on conotoxins. The complexity of their sequences, the broad diversity of their targets, and the inherent structural diversity of conotoxins present challenges for constructing more diversified and high-accuracy classification and prediction models [39,40]. Additionally, the integration of multi-modal data, such as structural information and post-translational modifications, remains underexplored. These gaps highlight the need for more advanced computational approaches and comprehensive databases to accelerate conotoxin discovery and application.

**Figure 1:**
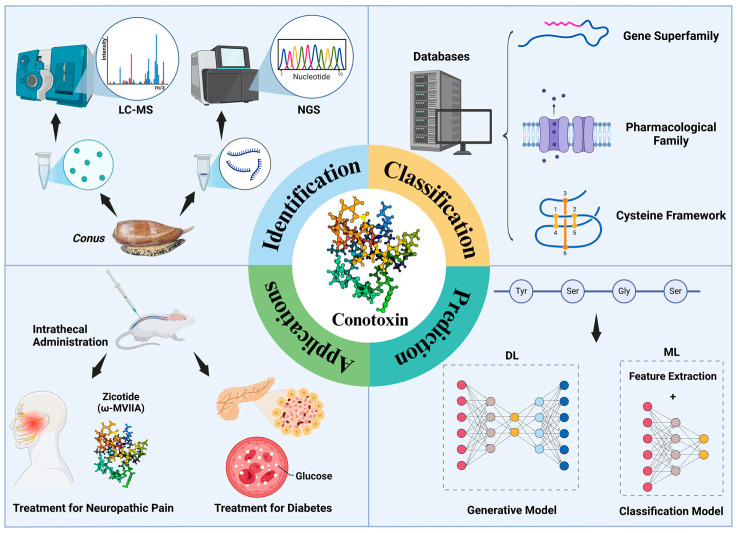
A schematic diagram of the content covered in this review. Conotoxin research encompasses four major aspects: (1) Identification, which has progressed from traditional chemical methods (e.g., MS) to high-throughput genomic and transcriptomic technologies, significantly improving discovery efficiency. (2) Classification, where conotoxins are grouped based on evolutionary superfamilies, pharmacological families, and cysteine frameworks. (3) Application, highlighting their therapeutic potential, such as intrathecal drug development for pain relief and diabetes treatment. (4) Prediction, where computational approaches, including machine learning, facilitate conotoxin sequence generation and classification. This figure summarizes the transition from traditional experimental techniques to modern computational bioinformatics in conotoxin research.

This review introduces the properties, classification, and applications of conotoxins, highlighting research in bioinformatics, including conotoxin databases, predictive models, and deep learning-based generation frameworks (Figure 1). It also provides an outlook on future research directions, such as database optimization, multi-modal data integration, and advanced modeling approaches.

## 2. Conotoxins

### 2.1. Sequence Characteristics of Conotoxins

Conotoxins are a group of neurotoxic peptides isolated from the venom of *Conus* species, characterized by 10 to 40 amino acid residues and a high content of disulfide bonds, exhibiting high selectivity and biological activity [5]. They serve as useful high-affinity ligands for various receptors and ion channels [41], including ion channels, G protein-coupled receptors (GPCRs), transporters, and enzymes [42,43]. One of the most notable features of conotoxins is the high abundance of cysteine residues in their sequences, which form disulfide bonds that stabilize the three-dimensional structure of the toxin and confer resistance to degradation [44].

The genes encoding bioactive venom components are characterized by accelerated evolution, and conotoxins may represent one of the fastest-evolving gene products known to date [10,45,46]. As a precursor for conotoxin biosynthesis, its transcript sequences consist of three regions: the endoplasmic reticulum (ER) signal peptide, the mature peptide region, and pre- or post-mature peptide region [41]. These regions are crucial in determining the toxin’s synthesis, functionality, and targeting properties [5].

Conotoxin sequences exhibit significant diversity across different species [47], with considerable variation in the order of the mature peptide region. Only a small subset of conotoxins are expressed in two or more *Conus* species [30]. In contrast, the ER signal peptide is highly conserved within specific subgroups and is typically used to cluster different conotoxins into corresponding gene superfamilies [42,48]. These conserved amino acid regions are directly associated with the biological activity of the toxin. They exert their effects by binding to ion channels in the nervous system, such as sodium, calcium, and potassium channels, or receptors, thereby inhibiting neurotransmission or modulating neural signaling [5].

Conotoxins typically undergo various post-translational modifications, many of which cannot be predicted from the precursor sequence. The formation of disulfide bonds is the most common post-translational modification in conotoxins [49]. In addition, most conotoxins carry additional post-translational modifications, such as hydroxylation of proline [49], sulfonation of tyrosine [50], and hydroxylation of glutamic acid [51]. These modifications further enhance the stability and biological activity of the toxins [52].

The structural and functional complexity of conotoxins is largely influenced by their post-translational modifications, which play a crucial role in defining their stability, bioactivity, and target specificity [50]. The formation of disulfide bonds, the most prevalent modification, is essential for maintaining the three-dimensional conformation of these peptides, thereby enhancing their resistance to enzymatic degradation and ensuring their biological function [53]. Additionally, modifications such as hydroxylation of proline, sulfonation of tyrosine, and hydroxylation of glutamic acid further refine their molecular interactions, enabling conotoxins to exhibit remarkable selectivity toward ion channels and receptors. These biochemical alterations not only contribute to their pharmacological potency but also add another layer of complexity to their classification and functional annotation [54,55]. With the advancement of high-throughput sequencing and bioinformatics approaches, researchers are now better equipped to explore the impact of modifications on conotoxin diversity and activity, enhancing our understanding of their evolutionary significance and potential applications in biomedical research. For example, next-generation sequencing (NGS) technologies like Illumina and PacBio sequencing enable the rapid sequencing of venom gland transcriptomes, revealing novel conotoxins and their associated modifications [10,56]. Transcriptome sequencing was used to uncover new conotoxins in *Conus betulinus*, demonstrating the power of these methods [57]. Bioinformatics tools such as ConoServer and ConoMode assist in annotating and classifying conotoxins [28,29,30,58], while molecular dynamics simulations (e.g., using GROMACS or AMBER) provide insights into how structural features influence conotoxin activity [59,60]. These technologies and approaches are essential for advancing conotoxin research and expanding their biomedical potential.

### 2.2. Discovery and Identification of Conotoxins

Since their initial discovery in the 1960s [61], research on conotoxins has evolved from traditional chemical analysis to modern genomics and bioinformatics, which significantly enhanced the efficiency and accuracy of their discovery and identification ability.

Initially, the discovery of conotoxins relied primarily on the extraction and isolation technology from *Conus* venom. Researchers isolated various peptide molecules through chemical analysis techniques such as high-performance liquid chromatography (HPLC) and mass spectrometry (MS) [7,9] and verified their toxicity through bioactivity assays, including cell-based cytotoxicity assays, enzyme inhibition assays, and receptor binding assays [62,63]. However, as the diversity and functional complexity of conotoxins increased, traditional chemical separation methods began to face limitations in terms of time and sample volume, making it difficult to meet the demands for high-throughput screening of large numbers of toxins.

To address this challenge, modern discovery and identification methods have shifted toward high-throughput genomic and transcriptomic technologies [64,65,66]. Advanced sequencing techniques and bioinformatics tools reduced the need to separate toxins from venom. They can help to reveal tens of thousands of previously unknown animal venom peptides and protein sequences, including over 20,000 conotoxin sequences [6,67]. As of 30 November 2024, the search result for “venom duct transcriptome” provided transcriptomic data from various species and taxonomic groups. There was a total of 126 entries related to venom duct transcriptomes. These entries represent a range of biological groups, including chordates, arthropods, and mollusks. Excluding the seven entries from the species *Raphitoma purpurea* (family Raphitomidae), all other entries originate from the family Conidae, covering 19 species within the genus *Conus*. In addition, a retrospective review by Jin et al. in 2019 documented the raw reads, accession numbers, and references for the venom gland transcriptomes of 30 *Conus* species [5].

These data provide valuable resources for marine venom gland functional studies, enabling researchers to perform further comparative analyses and functional investigations. By sequencing the genomes and transcriptomes of conotoxins, researchers can directly predict and identify toxin genes at the genetic level. This approach allows for the faster discovery of novel toxins and expands the range of toxins identified [3,68]. Compared to traditional venom extraction methods, genomic and transcriptomic-based discovery can significantly increase efficiency and offer strong technical support for systematically studying the diversity of conotoxins.

Currently, many studies utilize a combination of proteomics and transcriptomics to explore conotoxins in the venom of individual *Conus* species [69,70,71]. Next-generation sequencing (NGS) of venom gland transcriptomes provides an unbiased list of precursor sequences [5,42]. In addition to traditional proteomic techniques, advanced methods such as X-ray crystallography [72] and nuclear magnetic resonance NOESY [73,74]/ROESY [75] experiments are used to identify mature peptides and post-translational modifications.

The methods for identifying conotoxins have undergone a revolutionary transformation applying bioinformatics and computational biology techniques. Traditional identification methods rely on the separation and purification of venom, which often face challenges such as difficulties in sample acquisition (especially for rare and/or dangerous species) and are time-consuming [10]. Meanwhile, modern identification methods integrated high-throughput gene sequencing and bioinformatics tools, allowing for the direct prediction of toxins from genes and transcripts based on sequence information. Sequence alignment tools such as BLAST [76] and HMMER [77] have been used for identifying toxin genes. Compared with known toxins, these tools can quickly identify and validate new toxins [3,15,71,78]. Machine learning methods are widely employed in the identification and classification of proteins or peptides, considering features such as amino acid composition, n-mer amino acid composition, pseudo-amino acid composition, and position-specific scoring matrices (PSSM) [15,16,17,18,19,20,21]. These computational approaches not only provide new ways to rapidly and accurately identify conotoxins and their categories but also offer possibilities for a deeper understanding of their functional characteristics, overcoming the limitations of traditional biochemical experimental methods [79].

In conclusion, the discovery and identification of conotoxins have evolved from traditional chemical analysis to a combination of genomics, transcriptomics, and computational biology techniques. Modern genomic and bioinformatics methods have greatly enhanced the efficiency of toxin discovery and identification, providing support for the functional prediction, classification, and drug development of novel toxins.

### 2.3. Classification of Conotoxins

As research on conotoxins has advanced, these toxins have been classified into different cysteine frameworks, gene superfamilies, or pharmacological families based on factors such as the cysteine motifs in their mature peptide regions, the similarity of the precursor endoplasmic reticulum (ER) signal sequences, and their specificity for pharmacological targets [28,29,80]. The classification of conotoxins was essential for understanding their biological functions and provided a theoretical basis for drug development and neuroscience research.

#### 2.3.1. Cysteine Patterns of Conotoxins

A distinctive feature of conotoxins is their high content of cysteine residues. During the maturation process of proteins in eukaryotes, cysteine residues were oxidized to form disulfide bonds, which stabilize the three-dimensional structure of the protein [81,82]. The arrangement and number of disulfide bonds vary among toxins from different superfamilies and families, and these differences not only influence the stability of the toxin but also have significant effects on its binding affinity to targets and its toxicity [83]. The diversity in the patterns of disulfide bond connectivity allows for the classification of conotoxins based on their cysteine frameworks [80]. A cysteine framework was defined by the number of cysteine residues and the number of residues (either none or at least one) between adjacent cysteine residues [28,29]. There were 30 framework families classified in ConoServer based on the number of cysteine residues, their ring sizes, and the different disulfide bond linkages. A significant portion of these cysteine frameworks has been reported in transcriptomic studies.

#### 2.3.2. Superfamilies of Conotoxins

The classification of conotoxin superfamilies is based on the similarity of their signal peptide sequences, the arrangement of disulfide bonds, and their evolutionary relationships. Each superfamily encompasses a group of toxins that share conserved regions but exhibit functional diversity [5]. These toxins possess similar structures, and conserved signal peptide sequences. They target a wide range of receptors or ion channels within the nervous system.

As of the current understanding, the identified superfamilies of conotoxins have been divided into over 20 major categories, which can further subdivide into different branches based on the amino acid characteristics of their frameworks. Moreover, some conotoxins that have been characterized do not belong to any of the established gene superfamilies [80,84,85] and are classified into temporary gene superfamilies in the ConoServer database [28]. Well-known superfamilies, including A, M, P, O, S, T, and I, were associated with distinct biological functions and mechanisms of action. For instance, conotoxins from the A superfamily predominantly feature a type I cysteine framework (CC-C-C) and are known to effectively and selectively target a range of neuronal and neuromuscular nAChR subtypes [80]. Conantokin-G, from the B superfamily, is a non-competitive N-methyl-D-aspartate receptor (NMDAR) antagonist [86] and is currently under extensive investigation as a potential drug for pain relief, anticonvulsant therapy, and treatment of other neurological conditions [87,88,89]. The M superfamily, including branches M-4 and M-5, targets molecular sites such as voltage-gated potassium channels, acetylcholine receptors, and voltage-gated sodium channels, which can be further categorized into distinct pharmacological families [90,91].

In addition to the well-established superfamilies mentioned above, Robinson et al. also recorded four additional superfamilies not listed in the ConoServer database, namely ConoCAPs, Conopressins/Conophysins, Conkunitzins, and Con-Ikot-Ikots, as well as some conotoxins that have not yet been classified [80]. Table 1 summarizes the superfamilies mentioned above, along with their associated cysteine frameworks and pharmacological families. Scientists can further categorize these toxins into superfamilies or families utilizing machine learning methods in combination with sequence alignment and evolutionary analysis, thereby revealing their biological characteristics.

#### 2.3.3. Pharmacological Families of Conotoxins

Conotoxins are classified into numerous pharmacological families based on the type of their molecular targets and their corresponding pharmacological activities [1]. These toxins exhibit highly diverse structures and functions but share a common specificity for receptors. The primary targets of conotoxins are membrane proteins, particularly ion channels, membrane receptors, and transporters [92]. Conotoxins exert their biological effects by binding to various receptors or ion channels in the nervous system, modulating the transmission of neural signals and neuronal excitability [5]. Due to their ability to selectively interact with specific pharmacological targets, conotoxins are widely used as pharmacological tools in research related to disease treatment [93,94].

Conotoxins are widely used to study ion channel functions and their dysfunctions [67]. Ion channel-targeting conotoxins interact with specific ion channels, altering their permeability and regulating neuronal electrical activity. Based on the type of ion channel targeted, ion channel-specific conotoxins can be further categorized as follows:

(1) Sodium Channel-Targeting Toxins (Na-Conotoxins): μ-conotoxins act as classical pore blockers, binding directly to the sodium channel pore and inhibiting sodium ion flux [90,95]. δ-conotoxins and ι-conotoxins, on the other hand, function as gating modifiers, modulating the gating states of sodium channels, thereby affecting sodium ion flow and regulating the propagation of neural signals [53,96].
(2) Calcium Channel-Targeting Toxins (Ca-Conotoxins): ω-conotoxins block the passage of Ca^2+^ through voltage-gated calcium channels (CaVs) at presynaptic terminals, thereby disrupting the release of acetylcholine-containing vesicles and interfering with neurotransmission [97]. ω-MVIIA conotoxin is the only conotoxin approved by the FDA for the treatment of severe chronic pain [98,99,100].
(3) Potassium Channel-Targeting Toxins (K-Conotoxins): μ-conotoxin, although primarily known as a sodium voltage-gated channel inhibitor, also exhibits similar blocking effects on Shaker K channels [101] and mammalian Kv1.2 channels [102]. This result suggests that certain toxins can exert broad effects on different types of ion channels.

In addition to ion channels, some conotoxins can target neurotransmitter receptors and other membrane proteins [43]. These pharmacological families are documented in Table 2. For example, the α-conotoxin family interacts with nicotinic acetylcholine receptors (nAChRs) to modulate neurotransmitter release and neuronal plasticity, potentially offering neuroprotective effects [103]. ρ-conotoxins may regulate G-protein coupled receptor (GPCR) signaling through interaction with α_1_-adrenergic receptors (α_1_-AR) [104]. Members of the neurohypophyseal conotoxin family, such as contulakin-G (CGX-1160), act on GPCRs and play critical roles in neurotransmission and neuromodulation [105]. The diverse mechanisms of action of these toxins highlight their significant potential in neuropharmacological research and clinical applications, including the treatment of pain, epilepsy, anesthesia, and neuroinjury [106]. Classification based on their targets facilitates a deeper understanding of the biological functions of these toxins and provides a theoretical foundation for the development of targeted therapeutics [79].

### 2.4. Action and Applications of Conotoxins

**Figure 2:**
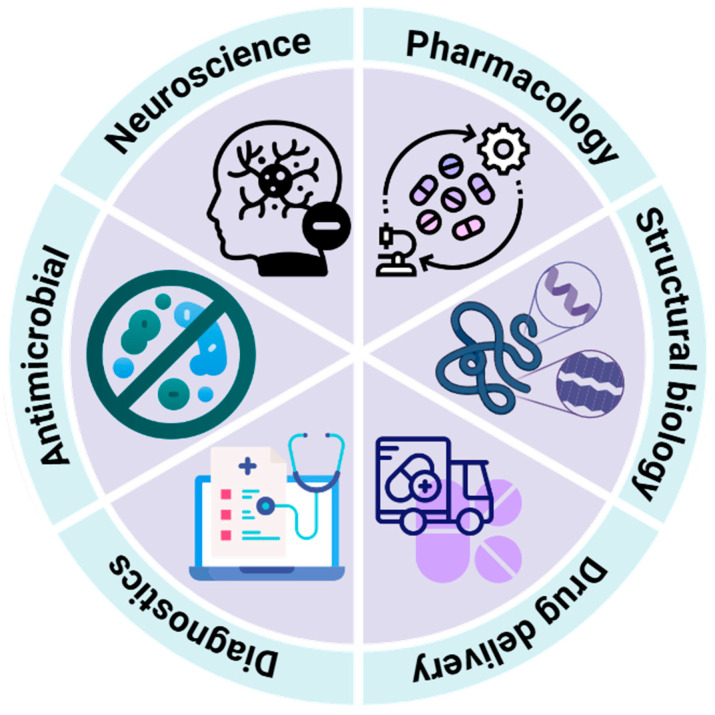
Key applications and potential of conotoxins. This figure highlights the diverse applications of conotoxins in various fields: (1) Pharmacology: Conotoxins are used in developing selective drugs for pain management, diabetes, and neurodegenerative diseases. (2) Neuroscience: By targeting specific ion channels and receptors, conotoxins regulate neurotransmission and cellular functions, advancing the study of neural signaling and circuits. (3) Antimicrobial: α-conotoxin RgIA mutants demonstrate antimicrobial activity, offering new opportunities for antibiotic development. (4) Diagnostics: Conotoxins are explored as novel diagnostic tools for early disease detection. (5) Drug Delivery and Anticancer Therapies: The α-ImI-paclitaxel conjugate shows promise in reducing tumor size and systemic toxicity, suggesting a new approach to cancer therapy. (6) Structural Biology: Conotoxins contribute to peptide biosynthesis research and receptor crystallization, supporting drug development and basic science.

Conotoxins, due to their structural stability, relatively small size, and excellent target specificity, serve as ideal molecular probes for target validation and peptide drug discovery [5]. Currently, conotoxins have become valuable research tools in neuroscience, pharmacology, biochemistry, structural biology, and molecular evolution (Figure 2). For example, in neuroscience, conotoxins are used to study neurotransmission [104,107]; in pharmacology, they are investigated as potential drugs, such as Prialt (ziconotide) for chronic pain [98,99]; in biochemistry, they aid in peptide biosynthesis studies [42,92,108]; and in molecular evolution, they are used to explore venom peptide evolution across *Conus* species [71,109]. They are also investigated as potential drugs, diagnostic agents, and drug leads [6]. By targeting specific ion channels, receptors, and other membrane proteins in the nervous system, conotoxins regulate neurotransmission and cellular functions, demonstrating broad potential for application in various fields.

Conotoxins hold significant potential in pain management, particularly in the treatment of neuropathic pain. Traditional pain management approaches, such as the use of opioid drugs, often lead to side effects such as addiction. In contrast, conotoxins, due to their high selectivity for specific ion channels, can effectively alleviate pain without the risk of addiction [4,6,99]. ω-conotoxin MVIIA (Ziconotide) is currently the only conotoxin-based drug approved by the FDA for refractory chronic pain. It exerts its effect by selectively blocking N-type calcium channels, thereby inhibiting neurotransmitter release and reducing the transmission of pain signals [98]. The clinical application of Ziconotide has demonstrated the unique advantages of conotoxins in pain management, particularly in patients who do not respond to conventional treatments [99]. Recent studies have indicated that the α-conotoxin RgIA derivative, RgIA4 (also known as KCP-400), acts as an antagonist of α9α10 nAChRs, showing high efficacy in both humans and rodents. Moreover, its selective inhibition of α9α10 nAChRs is at least 1000 times stronger than that of other drug targets, positioning it as a promising potential analgesic for the treatment of neuropathic pain [110,111,112]. The research on RgIA4 provides new directions for developing therapies for neuropathic pain, particularly for chronic pain induced by chemotherapy.

Several studies have pointed to animal venom peptides as pharmacological tools and potential therapeutic agents for diabetes [53,55,113], with some peptides directly acting as mimics of endogenous metabolic hormones. Others target ion channels expressed in pancreatic β-cells [6]. Con-Ins G1, an insulin peptide derived from the venom of the geographic cone snail (*Conus geographus*) [114], can activate both fish and human insulin receptors. It is the true monomeric insulin, lacking the C-terminal B-chain required for dimerization [55,115], and as such, it can act more rapidly to reduce blood glucose levels. Another conotoxin, Conkunitzin-S1 (Conk-S1), derived from the venom of the striped cone snail (*Conus striatus*) [116], inhibits Kv1.7 delayed rectifier currents, leading to increased insulin secretion in rats following glucose stimulation of the pancreas. Unlike K_ATP channel blockers, such as glibenclamide, Conk-S1 does not induce hypoglycemia, and its effects on blood glucose are temporary, without altering basal glucose levels [55,117]. These characteristics make Conk-S1 a valuable tool for studying the role of Kv1.7 in insulin secretion and highlighting its potential as a treatment for type 2 diabetes (T2D).

Conotoxins, through their binding to ion channels on neuronal membranes, precisely regulate neuronal excitability, neurotransmitter release, and membrane potential changes. This ability has positioned them as powerful tools for investigating neuroelectrophysiology, signal transduction, and neural circuits [83,93]. It also can provide a theoretical foundation for the treatment of neurological disorders. These toxins have demonstrated significant potential in the treatment of neurodegenerative diseases, such as Alzheimer’s disease and Parkinson’s disease [93]. They can selectively modulate inter-neuronal communication and enhance synaptic function. Furthermore, the high specificity and adjustable structure of conotoxins make them ideal candidates for novel therapeutic agents for neurodegenerative conditions.

The potential of conotoxins in fields such as antimicrobial [118,119] and anticancer [99,120,121] therapies is gradually being explored, revealing broad application prospects. For instance, a study demonstrated that α-ImI-conjugated paclitaxel, a chemotherapeutic drug, significantly reduced tumor mass in mice compared to the unconjugated control group while also decreasing systemic toxicity, highlighting the potential of conotoxins in drug delivery [120]. Additionally, research involving the synthesis of nine α-conotoxin RgIA mutants using D-amino acids revealed their antimicrobial activity against pathogens and fungi by disrupting bacterial cell membranes. These mutants exhibited low hemolytic activity, reduced cytotoxicity, and enhanced stability, providing a novel approach to antibiotic development [119]. Furthermore, α-conotoxins enhance the antitumor effects of cyclooxygenase and lipoxygenase inhibitors in Ehrlich carcinoma models in vitro and in vivo [122]. PnIA, RgIA, and ArIB11L16D potentiate the cytotoxic activity of baicalein and nordihydroguaiaretic acid, leading to reduced tumor growth and improved survival rates in mice [122]. These findings highlight the therapeutic potential of α-conotoxins in cancer treatment.

As peptide molecules, conotoxins still face challenges commonly associated with peptide-based drugs. Currently, conotoxin-based pharmaceuticals on the market require intrathecal injection for direct delivery into the spinal cord, an invasive method. Therefore, the development of alternative, less invasive delivery methods, such as oral administration, represents a more ideal approach [94]. However, conotoxins possess several unique properties, including small molecular size, chemical diversity, and post-translational modifications, which offer considerable promise for both clinical and research applications. These features provide strong support for drug development and foundational scientific research. In addition, conotoxins have diverse roles across various fields, including medical diagnostics, structural biology, cosmetics, and agriculture, further highlighting their broad potential [6].

## 3. Conotoxin Databases and Resources

Conotoxin databases and resources play a crucial role in the field of bioinformatics, making significant contributions to sequence information retrieval, functional annotation, sequence analysis, evolutionary and phylogenetic studies, drug design and screening, as well as data sharing and collaboration. Traditional protein databases, such as UniProtKB, the Protein Data Bank, the Biological Magnetic Resonance Data Bank, and the protein databases provided by the National Center for Biotechnology Information (NCBI), are primary sources of molecular biology data. In previous studies, researchers have frequently utilized these resources to gather relevant information.

ConoServer (https://www.conoserver.org/, accessed on 26 December 2024), a specialized database for collecting conotoxin sequence and structural information, provides a wealth of data on conotoxins, making it a valuable resource for conotoxin research [28,29]. As of 1 December 2024, ConoServer has curated 3073 nucleotide sequences (from 90 *Conus* species), 8523 protein sequences (from 123 *Conus* species), and 247 3D structures (representing 14 cysteine frameworks from 48 *Conus* species). ConoServer organizes its data into a detailed classification scheme based on gene superfamilies, cysteine frameworks, and pharmacological families. These classifications, along with sequence annotations, provide an extensive set of features (such as amino acid composition, sequence motifs, and conserved regions), which are crucial for training machine learning models focused on peptide sequence analysis. The regularly updated data in ConoServer aid in the creation of high-quality datasets for classification and prediction and has been widely used to predict the classes and properties of conotoxins, including toxicity, ion channel targeting, and receptor binding.

The quantities of various classes in benchmark datasets based on different classification criteria, along with the associated references, are presented in Table 3.

The first benchmark dataset for conotoxin superfamilies, called S1, includes 116 mature conotoxin sequences, along with a negative dataset containing 60 short peptide sequences that do not belong to any of the four superfamilies (A, M, O, or T) [123]. The second benchmark dataset, S2, expands upon this by including 261 entries from SwissProt that cover the four superfamilies, providing a broader and more diverse resource for classification tasks [17].

In addition to gene family-based datasets, functional datasets focusing on ion channel-targeting conotoxins have also been developed. The non-redundant benchmark dataset I1 includes 112 mature conotoxins, comprising 24 K-conotoxins, 43 Na-conotoxins, and 45 Ca-conotoxins [125]. The I2 dataset provides an extended set of ion channel-targeting conotoxins with 145 sequences [128]. These functional datasets are all curated from UniProt.

Many studies have constructed additional conotoxin datasets through rigorous filtering and management steps to align with the classification objectives of the models, including the classification of gene superfamilies [21,22], prediction of novel conotoxins [22,23,24] and prediction of conotoxin classes and conotoxins that target nAChRs [40], as illustrated in Table 4.

## 4. Sequence-Based Classification Prediction and Generation Research

### 4.1. Classification Tools

In recent years, researchers have integrated transcriptomics, proteomics, biochemistry, physiology, and bioinformatics into the field of venomics to accelerate the discovery of conotoxins [1]. Venomics involves the prediction and classification of putative conotoxins at the transcriptomic level. Traditionally, this process has relied on time-consuming BLAST [76] searches of specialized databases, but homology-based searches are not always accurate in predicting the activity and function of toxins, often resulting in incomplete or false-positive results. As a complement to BLAST, tools such as ConoPrec [28] provide relevant hints for superfamily and family classification based on the signal peptide sequence of the submitted precursor. However, these tools still face issues when signal sequences are missing, making predictions impossible. With the expansion of machine learning applications in protein classification, several tools that combine machine learning methods have been developed to address these limitations.

#### 4.1.1. Conotoxin Superfamily Classification Models

In 2017, Dao et al. [79] systematically reviewed the progress of machine learning in the classification of conotoxins and provided a detailed description of the entire classification workflow. In the early stages of applying machine learning to conotoxin research, classification models were primarily focused on predicting conotoxin superfamily types. Most of these models were introduced in their review, though there has been relatively less research in this area in recent years. This paper highlights the updated version of ConoDictor 2.0, along with other classifiers that also use Profile Hidden Markov Models (pHMM) to capture sequence characteristics and compare their performance.

The Profile Hidden Markov Model (pHMM) offers a flexible probabilistic framework that can capture changes in sequences such as insertions and deletions [77]. By training on multiple sequence alignments and utilizing known sequence data to construct the model, pHMMs can identify both conserved and variable regions within sequences, making them well-suited for representing biological sequences with diverse and complex structures [129]. The effectiveness of this method in capturing sequence features of other proteins has been well-established [130,131,132]. Silja Laht et al. [15] developed a computational method based on pHMM to predict and classify all described conotoxin superfamilies and families to identify new conotoxins from *Conus* genomes or transcriptomes. They constructed independent models based on 24 described conotoxin categories (16 superfamilies and 8 families), focusing on signal, propeptide, and mature regions. This approach overcame the issue of signal peptide absence and ultimately led to the development of 62 pHMM models, most of which demonstrated high sensitivity and specificity even when trained on small sample sizes.

ConoSorter is a sequence pattern search and discovery pipeline based on regular expressions and pHMMs [21]. The process begins with a stringent screening using regular expressions to filter out sequences that match known patterns. Then, the pHMM is employed to validate and identify more complex sequences that were mismatched initially. This hierarchical filtering strategy allows ConoSorter to perform an initial classification of sequences and then refine the analysis of edge sequences using more complex models. This approach can improve computational efficiency and enhance the capability to handle large-scale datasets. Additionally, ConoSorter offers a significant advantage in discovering new toxins compared to previous tools. However, the program still requires substantial manual input from users for result classification, especially when dealing with novel or atypical sequences.

ConoDictor combines two complementary feature-capturing methods, the Position-Specific Scoring Matrix (PSSM) and Profile Hidden Markov Model (pHMM), to build models for classification and prediction [22,133]. PSSM captures evolutionary characteristics within protein sequences by quantifying both their conserved and variable regions, helping to reveal evolutionary relationships, conserved functional domains, and evolutionary variations within protein families [134]. This method based on multiple sequence alignments of homologous sequences can calculate the frequency of each amino acid or nucleotide at each position and compare it with the background frequency to generate a score matrix. The matrix used to assess the similarity between a new sequence and conserved features is particularly useful for identifying conserved regions and patterns within the sequence [135,136].

ConoDictor enhances prediction accuracy by merging the match lists of pHMM and PSSM, applying weights based on the frequency of each predicted feature. The final classification result corresponds to the superfamily with the highest frequency. PSSM rapidly identifies conserved sequence patterns, which aids in initial screening and classification. On the other hand, the probabilistic framework of pHMM is more adept at capturing changes such as insertions or deletions in complex sequences, allowing for more precise classification. Therefore, the integration of both methods significantly improves classification sensitivity and specificity, particularly in cases where sequence data are incomplete or exhibit high variability.

The recently published ConoDictor 2.0 is an optimized and updated version of the original tool, providing an independent command-line utility with enhanced prediction accuracy and faster analysis speed [22]. The original version of ConoDictor [133] was limited by being web-based, which included potential bandwidth constraints and reduced flexibility for handling large datasets. The upgraded ConoDictor 2.0 addresses these limitations by supporting an offline, independent command-line interface along with multicore processing capabilities, providing improved prediction accuracy and faster analysis speeds. This upgrade lifts the restrictions on handling large transcriptome or proteome datasets, making it sufficiently capable of meeting the demands of large-scale *Conus* transcriptome or proteome analyses.

In terms of model training and optimization [137,138], the authors created a new benchmark dataset to eliminate erroneous annotations. This dataset was derived from UniProt, excluding the Conoserver data used in previous model training and retaining only complete precursors with superfamily annotations. The final dataset contained 727 sequences. The number of models in ConoDictor 2.0 increased from 48 in the original version to 158, covering more conotoxin superfamilies and variants. These models encompass known superfamilies and introduce new classifications. Therefore, ConoDictor 2.0 is better suited to adapt to highly complex sequences, such as those belonging to newly discovered superfamilies of conotoxins. Additionally, this version utilizes an optimized set of PSSM and pHMM, enabling efficient processing of the entire venom duct transcriptome while reducing false positives. By improving model structure and training data, ConoDictor 2.0 provides greater accuracy in handling sequence variability and incompleteness. It links identified conotoxins to the correct gene superfamily with an efficiency of nearly 99%. The establishment of multiple specific models for a single family significantly enhances specificity.

#### 4.1.2. Ion Channel-Targeted Conotoxin Classification Models

The classification of conotoxins into superfamilies can only provide indirect insights into their potential functions and cannot predict their specific receptor targets. For instance, both Δ-conotoxin-like Ac6.1 and Ω-conotoxin-like Ai6.2 belong to the O1 superfamily but target different types of ion channels. The Δ-conotoxin-like Ac6.1 interacts with voltage-gated sodium channels, while the Ω-conotoxin-like Ai6.2 inhibits voltage-gated calcium channels [139]. As a result, numerous machine learning-based prediction tools have emerged in this field, with detailed information on methods provided in Table 5.

Yuan et al. were the first to construct a non-redundant benchmark dataset I1 and predict the types of conotoxins targeting ion channels [125]. Using the dipeptide composition as a feature and optimizing the feature set based on the binomial distribution, they employed a radial basis function network to predict the types of conotoxins that target ion channels.

Subsequently, a series of studies were conducted building on this foundation, with most machine learning methods utilizing benchmark datasets for model training. Both iCTX-Type [126] and the model used pseudo-amino acid composition (PseAAC) [127] as a feature. They applied F-score-based algorithms for feature selection, with support vector machines (SVM) employed as the classification algorithm. However, three new amino acid residue properties, including rigidity, flexibility, and irreplaceability, were incorporated into the PseAAC. Their approach outperformed previous methods overall.

Two additional models based on this benchmark’s dataset had progressed in feature selection and optimization. Analysis of Variance (ANOVA) was employed to classify ion channel-targeted conotoxins [140] to select the best features from the 400-dimensional dipeptide composition. It can calculate the ratio between-group to within-group variance for each attribute with a strong statistical foundation that allows for direct testing of feature differences. A study published in 2020 developed a model named ICTC-RAAC, which similarly used N-peptide combinations (including amino acid composition, dipeptide, and tripeptide combinations) to represent conotoxin sequences. This model employed ANOVA combined with Incremental Feature Selection (IFS) to enhance prediction performance and help select the discriminative features based on their F-values. It improved network predictors and introduced a Reduced Amino Acid Cluster (RAAC) approach to simplify protein complexity [141,142]. RAAC clustered the 20 naturally occurring amino acids into groups based on their similar physicochemical properties, resulting in a simplified alphabet [143,144,145]. By reducing protein complexity, this method helps identify conserved sequence regions within proteins (Table 6).

The major models employ Support Vector Machines (SVM) with a Radial Basis Function (RBF) kernel as the classification algorithm. Support Vector Machines (SVM), initially developed by Vapnik et al. [146], are especially well-suited for small sample sizes, high-dimensional data, and complex pattern recognition tasks. SVM improves classification accuracy by maximizing the margin between classes and it can effectively avoid overfitting even with small datasets. It has been used in many practical applications, such as image recognition and text classification. The hierarchical use of SVM allows for the effective decomposition and resolution of complex classification tasks. Therefore, several studies have employed SVM to predict conotoxin types. The basic principle of SVM is to transform the input vectors into a high-dimensional Hilbert space and find a separating hyperplane in that space. The Gaussian Radial Basis Function (RBF) kernel [147,148] is a widely used kernel function due to its high performance in non-linear classification tasks.

A machine learning model, ICTCPred [128], was built based on I2 datasets for related predictions. The model uses hybrid features rather than a single discrete model to describe conotoxin samples. These hybrid features include 246 physicochemical properties of residues, obtained from APDbase, and incorporate composition, transition, and distribution (CTD), g-Gap dipeptide combinations (g-Gap DC), physicochemical properties (PP), and secondary structure information (SSI).

A notable challenge with this dataset is the imbalance among the different ion channel-targeting conotoxins, with Na^+^-channel-targeting conotoxins outnumbering Ca^2+^-channel-targeting conotoxins. To address this imbalance, ICTCPred employs the SMOTE technique (Synthetic Minority Over-sampling Technique) to balance the dataset, ensuring that the number of Na^+^-channel-targeting conotoxins is comparable to that of Ca^2+^-channel-targeting conotoxins.

Random Forest is a popular ensemble learning method applied to a wide range of biological prediction tasks [135,149,150,151,152,153]. It is capable of handling high-dimensional, complex, and noisy data and is effective in addressing issues with imbalanced datasets. By aggregating the results of multiple decision trees, Random Forest generates highly accurate classifiers, providing reliable predictions. In biology and medicine, it has proven to be an effective tool for disease prediction, gene analysis, and protein function prediction [154,155,156,157,158].

#### 4.1.3. Toxicity Prediction Models

It is estimated over 700 species of *Conus* worldwide [159], each capable of secreting more than 1000 distinct conotoxins [1]. The total number of different bioactive peptides produced by *Conus* is estimated to be around one million. However, to date, fewer than 0.1% of these conotoxins have been fully characterized [5,93]. Therefore, the discovery of novel conotoxins from the vast reservoir of these compounds using high-throughput and sensitive methods is crucial for the development of *Conus*-based pharmaceuticals.

Traditional methods for peptide toxicity prediction, including crude venom purification, cloning of conotoxin precursor genes, venom duct transcriptomics, and venom proteomics, often rely on experimental laboratory techniques. These methods are not only time-consuming and labor-intensive but also costly [3]. In contrast, machine learning provides an automated, efficient, and precise solution that makes the toxin discovery process faster, more accurate, and scalable.

In recent years, several tools have been developed through deep learning or machine learning (ML) techniques to predict the toxicity of peptides based on their primary amino acid sequences, thereby improving the toxicity identification process. The tools, such as ClanTox [160], ToxClassifier [23], ToxPred [161], and TOXIFY [162], are constructed to distinguish venom proteins from non-venom proteins, offering valuable insights into conotoxin recognition. Published conotoxin identification models, such as ConusPipe [23] and ConoDictor 2.0 [22], have significant improvements in the machine learning models and feature selection to some extent enhanced prediction accuracy.

ConusPipe utilizes the chemical features of conotoxins to predict toxin transcripts in the *Conus* transcriptome, overcoming the limitations of traditional homology-based search methods (such as BlastX [28,29] and HMMER [21,163]). Even conotoxins that evolve rapidly and exhibit significant sequence divergence retain three conserved regions: the N-terminal signal sequence, the pro-peptide region, and the mature toxin region [8,164,165]. Based on these characteristics, ConusPipe constructs machine learning models and extracts 16 features (including SignalP D-value, cysteine percentage, molecular weight, the percentage of positively and negatively charged amino acids, and isoelectric point) from 4950 known toxin sequences and 52,613 non-toxic transcripts. Given the limited training data and a large dataset, three machine learning models are employed: logistic regression [166], effective in binary classification; semi-supervised learning (label spreading), which leverages unlabeled true positives; and artificial neural networks (perceptron), which perform well on large training sets, and are combined with cross-species Blastp to prevent the omission of potential toxin sequences.

When tested on 540,261 protein sequences, the ConusPipe model combination achieved a specificity of 99.92% in distinguishing conotoxins from other proteins. After performing homologous sequence filtering with the Uniprot and ConoServer databases, the remaining transcripts were input into the model for prediction, identifying new candidate toxins. Ultimately, from the further filtered 5148 sequences, 187 were recognized by all four models, and 709 were recognized by three models, validating the effectiveness and advantage of the multi-model combination in toxin discovery.

The tools mentioned in the previous section, Conosorter [21,78] and ConoDictor 2.0 [22], are primarily designed to classify conotoxins from different superfamilies, but they can also identify whether a sequence is a conotoxin. ConoDictor was tested using a dataset containing known, correctly annotated conotoxins, along with sequences from random toxins (spiders, snakes, sea urchins, jellyfish) from UniProtKB. The results showed that ConoDictor 2.0 outperforms ConoSorter v1.1 and BLAST v2.9.0+ in distinguishing conopeptides, with the highest specificity.

Conotoxin proteins typically feature flexible peptide backbones, a high proportion of cysteine-linked disulfide bond patterns, and rich post-translational modifications, characteristics that pose significant challenges for toxin prediction. Most current prediction methods and models neglect the impact of disulfide bond patterns and certain post-translational modifications on the prediction outcomes. In recent research, integrated physicochemical features (P), secondary structure features (SS), post-translational modification (P2) and collision cross-section (CCS) [24] significantly enhanced the model’s performance in distinguishing between the toxin and non-toxic peptides. Among these, the collision cross-section (CCS) is a novel feature introduced for conotoxin prediction. CCS values, derived from ion mobility spectrometry (IM-MS) experiments or high-performance collision cross-section software (HPCCS) [167], represent experimental measurements that depend on factors such as molecular size, shape, charge, and polarization. The inclusion of this feature supplements the missing three-dimensional structural information and molecular interaction characteristics in existing conotoxin prediction methods. The CCS reflects the conformational differences between isomers of the same chemical composition formed by different disulfide bonds in conotoxins, thus aiding in the assessment of their varying toxicities.

Additionally, the authors classified negative samples into “easy negatives” (non-toxic samples) and “hard negatives” (toxic peptides from other species), and used a combination of machine learning models—penalized logistic regression (PLR), support vector machine (SVM), random forest (RF), and XGBoost—for training, which improved the model’s ability to distinguish complex negative samples. By employing multi-level sample classification and integrating diverse features, Monroe et al. significantly enhanced the accuracy of their model. In particular, random forests demonstrated excellent performance across multiple datasets, with higher overall accuracy (OA) and recall rates than the best existing method in the field, ToxinPred [161], especially in distinguishing between toxin and non-toxin proteins. When all features (P + SS + CCS + P2) were combined, all four classifiers performed optimally, with a converged OA of approximately 96%, an AA of 95%, and an F1 score of 0.92. For “hard negatives”, the model exhibited high robustness and precision, making the identification of conotoxins more reliable. Monroe et al.’s research, through the introduction of novel features and improved classification strategies, successfully achieved precise conotoxin prediction in complex contexts, laying a foundation for further understanding their biological functions and potential in drug development.

Overall, from the integrated classification capabilities of ConusPipe and ConoDictor 2.0 to the novel features and advanced classification models introduced by Monroe et al., significant breakthroughs have been made in the accuracy and applicability of conotoxin identification and classification methods. These studies provide robust tools and a solid theoretical foundation for large-scale toxin screening and future drug development. Additionally, toxicological testing methods, such as haemolytic assays and in vivo toxicity models, serve as valuable experimental validations for these prediction models. These traditional assays help assess the accuracy of the models by confirming whether peptides predicted to be toxic exhibit cytotoxicity [168,169], cause disruption to red blood cell membranes [104,168,169], or induce systemic toxicity in animal models [169]. For models that predict conotoxins, validation is further achieved through techniques such as mass spectrometry (MS), transcriptomics, proteomics, and structural biology analyses [74,92,170]. These methods ensure that the predicted peptides are conotoxins by confirming their identity and structural features. By integrating these experimental approaches with computational models, the accuracy of toxicity predictions can be significantly enhanced [171], providing a more reliable foundation for the identification and development of therapeutic agents.

### 4.2. Deep Learning-Based Generation and Prediction Framework

In recent years, deep learning (DL) has made significant advances in molecular generation and prediction. Tools such as ProteinGAN [172], ProGen [173], ProtGPT [174], and ProteinMPNN [175] have achieved tremendous success in generating proteins and peptides. Compared to traditional methods for discovering conotoxins, such as purifying toxins from venom or modifying toxin structures, deep learning approaches leverage large-scale data to learn and generate novel toxin molecules, greatly accelerating the discovery of new toxins. Against this backdrop, the recently proposed ConoDL framework [176] represents a breakthrough in this field. ConoDL consists of two core modules: the Conotoxin Generation Model (ConoGen) and the Conotoxin Prediction Model (ConoPred), which provide innovative tools and methods for the study and application of conotoxin.

ConoGen is based on the Transformer architecture and utilizes transfer learning techniques, employing the large language model ProGen to generate conotoxins. The Transformer model is a pivotal architecture in modern deep learning, widely applied across various fields such as language understanding, image processing, and information retrieval. Its core feature, the self-attention mechanism, connects all tokens in a sequence through a correlation-based pooling operation [177,178]. Due to its inherent advantages in processing sequential data, the Transformer model effectively captures the sequence characteristics of biological molecules like DNA, RNA, and proteins. While its attention mechanism helps identify long-term dependencies within complex molecular structures [178]. In ConoGen, the Transformer architecture is employed to learn the intricate internal patterns of conotoxins and generate artificial sequences resembling natural conotoxins. The successful application of this approach significantly enhances the generation and prediction capabilities of conotoxins.

ConoPred, based on the Wasserstein Autoencoder (WAE), was used for the prediction and screening of generated conotoxins, effectively removing sequences that are unlikely to be functional toxins and thus further improving the efficiency of the research process. The WAE model typically constrains the output of the encoder using a distribution, such as the standard normal distribution, ensuring that the representations in the latent space align. This constraint is achieved by minimizing the Wasserstein distance between the encoder’s output distribution and the distribution, enabling the model to distinguish which sequences are close to the data distribution and which deviate from the normal range [179]. As a result, the WAE model effectively captures the semantic relationships between peptide sequences, accelerating the discovery of functional peptides. It has been successfully applied to study antimicrobial peptides (AMPs) [180] and anticancer peptides (ACPs) [181]. The WAE plays a central role in feature extraction and generating probability scores. Its main function is to represent the input conotoxin sequences in the latent space and learn how to effectively encode and decode these sequences through reconstruction loss and prior constraints. Ultimately, the model uses this latent representation to assess whether the input sequence possesses features similar to real conotoxins, generating a probability score that indicates the likelihood of the sequence being a genuine conotoxin.

Experiments have demonstrated that the artificial conotoxins generated by ConoDL are like natural toxins in both sequence and spatial structure, and they exhibit novel cysteine scaffolds. These findings hold promise for advancing the molecular space exploration of conotoxins and the discovery of drug-active variants.

Emerging deep-learning techniques show significant potential in protein classification [182,183] and generation tasks [184,185]. Methods such as diffusion models [186], Generative Adversarial Networks (GANs) [187], Conditional Variational Autoencoders (CVAE) [188], multimodal Transformers [189], and Graph Neural Networks (GNNs) [190] have demonstrated advantages in generating diverse toxin sequences, enhancing prediction accuracy, and performing feature learning. The integration of generation and optimization frameworks with reinforcement learning is expected to lead to breakthroughs in conotoxin research. These methods not only improve the efficiency of new toxin discovery but also provide strong technical support and development potential for the application of toxins in drug development and neuroscience.

## 5. Future Research Directions

### 5.1. Optimization and Expansion of Data Resources

Current databases, such as ConoServer, have significantly contributed to conotoxin research by providing extensive sequence and structural information [28,29]. However, significant gaps remain in the coverage of rare superfamilies and novel variants of conotoxins [21,80]. Many of these rare or less-studied conotoxins have not been fully characterized or incorporated into existing databases, limiting the potential for comprehensive analysis and discovery. Additionally, the rapid evolution and high diversity of conotoxin sequences pose challenges for maintaining up-to-date and accurate databases [45,46].

The issue of constructing datasets is data imbalance. Certain conotoxins, such as those targeting sodium channels, tend to dominate the existing data, which may affect the accuracy of prediction models. Advanced sampling techniques have been employed to synthesize minority class samples, with some existing models already utilizing the SMOTE method to generate new samples [24,40,128]. Another approach involves using weighted loss functions to assign greater weight to minority class samples while integrating ensemble learning methods can effectively mitigate the problem of class imbalance [191,192].

Furthermore, the function of conotoxins is influenced not only by their amino acid sequences but also by factors such as their three-dimensional structures and post-translational modifications [193,194]. Future research could consider integrating sequence data with structural data (e.g., NMR, X-ray crystallography), mass spectrometry data, and other modal information to build more [28,29] robust hybrid models. For example, incorporating information about the three-dimensional structure of the toxins and their interactions with receptors into the model could significantly improve its ability to predict toxin function, particularly for functional prediction tasks, where structural data can provide crucial supplementary information.

### 5.2. Introduction of Multi-Modal Features

Current conotoxin classification and prediction models primarily rely on sequence information, such as amino acid composition and dipeptide patterns, with limited use of structural features and post-translational modifications [17,18,20,39,125,126,128,133,140]. Integrating multi-modal features, such as collision cross-section (CCS) and molecular surface charge distribution, could significantly enhance model performance. However, the development and application of such features in conotoxin research are still in their infancy. As our understanding of the mechanisms of action of conotoxins deepens, new features, such as collision cross-section (CCS), molecular surface charge distribution, and molecular similarity, are likely to play a key role in enhancing the performance of prediction models. For instance, the “collision cross-section” (CCS) [24,40] combined with mass spectrometry data successfully increased the distinction between toxins and non-toxins. In future research, further integrating these new features with multi-modal data (e.g., mass spectrometry, NMR) could significantly enhance the accuracy and generalizability of models.

### 5.3. Application of Deep Learning Methods in Conotoxin Bioinformatics Research

With the rapid development of deep learning technologies, particularly in protein generation and biomolecular research, deep learning has become a critical tool in bioinformatics [195,196]. In the realm of prediction, deep learning can address data scarcity through transfer learning and pre-trained models, significantly improving the accuracy of predictions [197,198,199] based on training on large datasets. While deep learning has shown great potential in protein generation and prediction, its application in conotoxin research is still limited. Current models often neglect the integration of structural biology and molecular dynamics simulations, which could provide crucial supplementary information for functional prediction. Future research should explore advanced deep learning techniques, such as multi-task learning and reinforcement learning, to improve conotoxin generation and classification. Deep learning models can automatically capture sequence characteristics and structural features of conotoxins, especially in complex toxins, allowing for the detection of subtle structural differences and the prediction of potential targets. Multi-task learning (MTL) has emerged to tackle the challenges of data diversity and structure complexity and enhance model adaptability and generalization. MTL has already shown promising results in various protein prediction models [200,201,202] and, when applied to conotoxins, can optimize models across multiple dimensions by jointly training tasks such as toxin type classification, function prediction, and toxicity assessment, thereby improving the ability to handle complex data.

In terms of generation, deep learning also exhibits immense potential. The algorithms, such as Generative Adversarial Networks (GANs) and Variational Autoencoders (VAEs), can discover new toxin molecules and explore uncharted molecular spaces. For instance, ConoDL, which employs a Transformer architecture to generate conotoxins, demonstrates the vast potential of deep learning in molecular generation [176]. These techniques, integrating known structural and functional data, not only enable the discovery of new toxin molecules but also facilitate the design of molecules with specific functions, providing possible candidates for new drug development.

### 5.4. Development of Diverse Models

Current classification tools primarily focus on ion channel targets, neglecting other potential targets such as nicotinic acetylcholine receptors (nAChRs) and G-protein coupled receptors (GPCRs) [39,125,126,127,140]. Additionally, quantitative structure–activity relationship (QSAR) models, which could predict the bioactivity and toxicity of new toxins, are underdeveloped. Future studies should expand to include multi-receptor target prediction and QSAR models to enhance the generalizability and accuracy of conotoxin research.

Although there has been progress in developing classification models for conotoxins targeting different ion channels, most research has focused on ion channel targets [39,125,126,127,140], neglecting other potential targets, such as nicotinic acetylcholine receptors (nAChRs), N-methyl-D-aspartate (NMDA) receptors, and G-protein coupled receptors (GPCRs). Conotoxins exert their effects by binding to these neurotransmitter receptors, modulating the transmission of nerve signals, and offering significant potential for their application in neuroscience and drug development. Some studies have begun to explore these non-ion channel receptors, such as predicting conotoxins that target acetylcholine receptors [24,40], but research in this area remains limited. Therefore, future studies should expand to include multi-receptor target prediction and classification models, using advanced algorithms such as multi-task learning and deep learning to identify the targeting of conotoxins to different receptors, thereby enhancing the generalizability and accuracy of the models. In addition, Raman spectroscopy combined with principal component analysis (PCA) has been explored for rapid and precise classification of conotoxins based on structural features, distinguishing between toxin types and even disulfide isomers [203]. With the accumulation of experimental data and the advancement of computational capabilities, integrating conotoxins structural biology, molecular dynamics simulations, and high-throughput screening technologies can further improve receptor-target classification models, providing a more accurate theoretical foundation for drug development and disease treatment applications.

Current classification tools primarily rely on extracting sequence information [204], often overlooking the spatial structure and functional characteristics. Given that conotoxins typically have complex three-dimensional structures and various post-translational modifications. Incorporating QSAR models will provide more precise information for toxin function prediction and drug design. QSAR is a predictive model based on the quantitative relationship between molecular structure and biological activity [205]. In QSAR models, the structural features of molecules (such as amino acid composition, disulfide bond patterns, molecular size, charge distribution, etc.) are statistically related to their biological activities (such as receptor affinity, inhibition, etc.), enabling the prediction of the activity of untested molecules [206,207]. For conotoxins, QSAR models can assist researchers in understanding how different structural features influence their interaction with specific targets. For example, some toxins act by binding to specific ion channels, while others may target acetylcholine receptors [42]. By constructing QSAR models, researchers can predict the potential targets and biological activities of unknown toxins, thus accelerating the discovery of novel drugs. Constructing high-quality QSAR models requires sufficient structural data and accurate biological activity data. With advances in structural biology techniques and the updating data resources, increasing numbers of conotoxin three-dimensional structural data are becoming available, providing the necessary structural information for QSAR modeling. Biological activity data typically include information on the toxin’s affinity for specific receptors, toxicity (e.g., LD50 values), neuroactivity (e.g., impact on neurotransmission), and inhibition of special effect ion channels. Currently, biological activity data are relatively scarce, while the related data through ongoing research will support the development of more robust QSAR models.

## 6. Conclusions

Conotoxins are potent peptides from cone snail venom known for modulating ion channels, receptors, and neurotransmitter systems. These molecules hold promise for drug development, especially in treating pain and neurological disorders.

We provide a detailed introduction to conotoxin research in bioinformatics, covering several key aspects: (1) Sequence Characteristics and Classification: Conotoxins feature unique cysteine-rich frameworks and disulfide bond patterns, essential for their function and classification. They are categorized into superfamilies and pharmacological families based on sequence homology and functional properties, aiding in understanding their mechanisms and therapeutic potential. (2) Databases and Resources: Significant progress has been made in creating conotoxin databases like UniProt and ConoServer, which provide extensive information on sequences, structures, and functions. However, gaps remain, particularly for rare or less studied toxin variants. (3) Machine Learning and Deep Learning in Conotoxin Research: Bioinformatics tools using machine learning (ML) and deep learning (DL) have advanced conotoxin research. Tools predict superfamilies and ion channel targets, while deep learning frameworks like ConoDL enable conotoxin sequence generation and improve function, toxicity, and receptor predictions.

As conotoxin research continues to evolve, several key areas hold promise for future advancements. These trends include the expansion of conotoxin databases, the incorporation of multi-modal data (e.g., structural and mass spectrometry data) into prediction models, and the application of advanced deep learning techniques [208,209,210] to improve conotoxin generation, classification, and function prediction. Furthermore, developing more diverse models such as protein language models [186,187] and deep forest models [188] for receptor prediction and quantitative structure–activity relationships (QSAR) will contribute to targeted therapies and drug discovery.

## Figures and Tables

**Table 1:** Classification of gene superfamilies and their associated cysteine frameworks and pharmacological families. The table includes only the established gene superfamilies and does not include temporary gene superfamilies. Data sourced from ConoServer and [80].

Gene Superfamily	Cysteine Framework	Pharmacological Family
A	I, II, IV, VI/VII, XIV, XXII	α, κ, ρ, other
B1		
B2	VIII	
B3	XXIV	α
C		α
D	IV, XIV, XV, XX, XXIV, XXVIII	α
E	XXII	
F		
G	XIII	
H	VI/VII	
I1	VI/VII, XI, XXII	ι
I2	VI/VII, XI, XII, XIII, XIV	κ
I3	VI/VII, XI	
J	XIV	α, κ
K	XXIII	
L	XIV, XXIV	α
M	I, II, III, IV, VI/VII, IX, XIV, XVI, XXXII	α, ι, κ, μ
N	XV	
O1	I, VI/VII, IX, XII, XIV, XVI, XXIX	δ, γ, κ, μ, ω
O2	I, VI/VII, XII, XIV, XV, XVI	γ
O3	VI/VII, XVI	
P	IX, XIV	
Q	VI/VII, XVI	
R	XIV	
S	VIII, XXXIII	α, σ
T	I, V, X, XVI	
U	VI/VII	
V	XV	
Y	VI/VII, XVII	
conodipine		
con-ikot-ikot	XXI,	
conoCAP		
conopressin		
conkunitzin	XIV	κ

**Table 2:** Pharmacological families used in ConoServer. This table summarizes the pharmacological families of conotoxins as classified in ConoServer. Each family is associated with specific molecular targets, including ion channels and receptors, and their representative toxins are listed for reference.

Family	Definition	Representative Toxin(s)
α (alpha)	Nicotinic acetylcholine receptors (nAChR)	GI
γ (gamma)	Neuronal pacemaker cation currents (inward cation current)	PnVIIA, TxVIIA
δ (delta)	Voltage-gated Na channels (agonist, delay inactivation)	TxVIA
ε (epsilon)	Presynaptic Ca channels or G protein-coupled presynaptic receptors	TxVA
ι (iota)	Voltage-gated Na channels (agonist, no delayed inactivation)	RXIA
κ (kappa)	Voltage-gated K channels (blocker)	PVIIA
μ (mu)	Voltage-gated Na channels (antagonist, blocker)	GIIIA
ρ (rho)	Alpha1-adrenoceptors (GPCR)	TIA
σ (sigma)	Serotonin-gated ion channels 5-HT3	GVIIIA
τ (tau)	Somatostatin receptor	CnVA
χ (chi)	Neuronal noradrenaline transporter	MrIA, CMrVIA
ω (omega)	Voltage-gated Ca channels (blocker)	GVIA

**Table 3:** The benchmark datasets of conotoxin superfamily and ion channel-targeted conotoxin [79]. This table summarizes the number of conotoxin sequences classified into various superfamilies and associated with specific ion channels. The data include both superfamily classification (A, M, O, T) and the types of ion channels targeted by conotoxins (K, Na, Ca), along with the total number of sequences available in each category.

**Superfamily**								
	A	M		O		T	Total Number	Reference
S1	24	43		45		17	116	[16,19,123,124]
S2	26	49		70		55	216	[17,18]
**Type of Ion Channel**								
	K-Conotoxin		Na-Conotoxin		Ca-Conotoxin		Total Number	Reference
I1	24		43		45		112	[39,125,126,127]
I2	26		49		70		145	[128]

**Table 4:** Additional conotoxin datasets. This table lists conotoxin-related datasets, including their source, number of sequences, main purpose, and reference. These datasets are used for tasks like superfamily classification and prediction of new conotoxins.

Model	Source	Dataset	Main Purpose	Reference
ConoSorter	ConoServer, UniProt	2008 sequences: 1390 from the conotoxin superfamilies and 1931 from the classes.	Superfamily Classification.	[21]
ConoDictor 2.0	UniProt	727 sequences (from 19 superfamilies)	Superfamily Classification and Prediction of New Conotoxins	[22]
ConusPipe	ConoServer, UniProt	4950 conotoxin sequence; 52,613 non-conotoxin transcripts	Prediction of New Conotoxins	[23]
	ConoServer, PDB, BMRB	Small P: 154; Extended P: 184 Small EN: 180; Extended EN: 560 Small HN: 178; Extended HN: 317	Prediction of New Conotoxins	[24]
	PDB, BMRB	102 nAChR binders (98 alpha/ 29 mu/21 omega and others) 82 non-nAChR binders	Predict conotoxin classes and conotoxins that target nAChRs	[40]

Table note: P: positive dataset contains conotoxins; EN: easy-negative dataset contains non-toxic peptides; HN: hard-negative dataset contains toxic peptides.

**Table 5:** Comparison of different methods for the type of ion channel-targeted prediction. This table compares the performance of different prediction methods for ion channel-targeted conotoxins, including K-conotoxins, Na-conotoxins, and Ca-conotoxins. The metrics used for evaluation are Sensitivity (Sn), Accuracy (AA), and Overall Accuracy (OA), as well as the corresponding datasets and references for each method.

Model	Feature Quantity	Feature ^a^	Feature Selection ^b^	Algorithm ^c^	Publication Year	Reference
RBF network	70	DPC	Binomial Distribution	RBF network	2013	[125]
iCTX-Type	50	PseAAC of DP Patterns	F-score, IFS	SVM (RBF Kernel)	2014	[126]
ICTCPred	503	Hybrid Features: CTD, g-Gap DC, PP, SSI	SMOTE, Relief, IFS	RF	2016	[128]
Fscore-SVM	180	PseAAC (newly added attributes)	F-score	SVM (RBF Kernel)	2016	[127]
AVC-SVM	68	DPC	ANOVA, PCC	SVM	2017	[140]
ICTC-RAAC	156	NPC (AAC/DPC/TPC)	RAAC, ANOVA, IFS	SVM (RBF Kernel)	2020	[39]

Table note: ^a^: AAC: amino acid composition; DPC: dipeptide composition; NPC: N-peptide combination; PseAAC: pseudo amino acid composition (newly added attributes:rigidity/flexibility/irreplaceability); CTD: Composition, Transition, Distribution features; G-gap DC: Gapped-Dihedral Correlation; PP: Physicochemical Properties; SSI: Secondary Structure Information. ^b^: IFS: Incremental Feature Selection; SMOTE: Synthetic Minority Over-sampling Technique; ANOVA: Analysis of Variance; PCC: Pearson Correlation Coefficient; RAAC: Reduced Amino Acid Composition. ^c^: RF: Random Forest; RBF: Radial Basis Function; SVM: Support Vector Machine.

**Table 6:** Comparison of performance for different types of ion channel-targeted prediction.

Methods	Dataset	Sn			AA	OA	Reference
		K-Conotoxin	Na-Conotoxin	Ca-Conotoxin			
RBF network	I1	0.917	0.884	0.889	0.897	0.893	[125]
iCTX-Type	I1	0.833	0.978	0.898	0.903	0.911	[126]
ICTCPred	I2	1	0.919	1	0.973	0.957	[128]
Fscore-SVM	I1	0.917	0.953	0.953	0.942	0.946	[127]
AVC-SVM	I1	0.931	0.942	0.892	0.922	0.920	[140]
ICTC-RAAC	I1	0.917	0.954	10		0.964	[39]

## Data Availability

No new data were created or analyzed in this study.

## References

[1] Dutertre S., Jin A.H., Kaas Q., Jones A., Alewood P.F., Lewis R.J. (2013). Deep venomics reveals the mechanism for expanded peptide diversity in cone snail venom. Mol. Cell. Proteom..

[2] Margiotta F., Micheli L., Ciampi C., Ghelardini C., McIntosh J.M., Di Cesare Mannelli L. (2022). *Conus* regius-derived conotoxins: Novel therapeutic opportunities from a marine organism. Mar. Drugs.

[3] Fu Y., Li C., Dong S., Wu Y., Zhangsun D., Luo S. (2018). Discovery methodology of novel conotoxins from *Conus* species. Mar. Drugs.

[4] Pennington M.W., Czerwinski A., Norton R.S. (2018). Peptide therapeutics from venom: Current status and potential. Bioorganic Med. Chem..

[5] Jin A.H., Muttenthaler M., Dutertre S., Himaya S.W.A., Kaas Q., Craik D.J., Lewis R.J., Alewood P.F. (2019). Conotoxins: Chemistry and biology. Chem. Rev..

[6] Bjørn-Yoshimoto W.E., Ramiro I.B.L., Yandell M., McIntosh J.M., Olivera B.M., Ellgaard L., Safavi-Hemami H. (2020). Curses or cures: A review of the numerous benefits versus the biosecurity concerns of conotoxin research. Biomedicines.

[7] Cruz L.J., Gray W.R., Olivera B.M. (1978). Purification and properties of a myotoxin from *Conus geographus* venom. Arch. Biochem. Biophys..

[8] Bandyopadhyay P.K., Colledge C.J., Walker C.S., Zhou L.M., Hillyard D.R., Olivera B.M. (1998). Conantokin-G precursor and its role in gamma-carboxylation by a vitamin K-dependent carboxylase from a *Conus snail*. J. Biol. Chem..

[9] Gray W.R., Luque A., Olivera B.M., Barrett J., Cruz L.J. (1981). Peptide toxins from *Conus geographus* venom. J. Biol. Chem..

[10] Phuong M.A., Mahardika G.N. (2018). Targeted sequencing of venom genes from cone snail genomes improves understanding of conotoxin molecular evolution. Mol. Biol. Evol..

[11] Charoenkwan P., Schaduangrat N., Manavalan B., Shoombuatong W. (2025). M3S-ALG: Improved and robust prediction of allergenicity of chemical compounds by using a novel multi-step stacking strategy. Future Gener. Comput. Syst..

[12] Charoenkwan P., Schaduangrat N., Pham N.T., Manavalan B., Shoombuatong W. (2023). Pretoria: An effective computational approach for accurate and high-throughput identification of CD8+ t-cell epitopes of eukaryotic pathogens. Int. J. Biol. Macromol..

[13] Shoombuatong W., Homdee N., Schaduangrat N., Chumnanpuen P. (2024). Leveraging a meta-learning approach to advance the accuracy of Nav blocking peptides prediction. Sci. Rep..

[14] Shoombuatong W., Meewan I., Mookdarsanit L., Schaduangrat N. (2024). Stack-HDAC3i: A high-precision identification of HDAC3 inhibitors by exploiting a stacked ensemble-learning framework. Methods.

[15] Laht S., Koua D., Kaplinski L., Lisacek F., Stöcklin R., Remm M. (2012). Identification and classification of conopeptides using profile hidden markov models. Biochim. Et Biophys. Acta BBA Proteins Proteom..

[16] Lin H., Li Q.Z. (2007). Predicting conotoxin superfamily and family by using pseudo amino acid composition and modified mahalanobis discriminant. Biochem. Biophys. Res. Commun..

[17] Fan Y.X., Song J., Kong X., Shen H.B. (2011). PredCSF: An Integrated Feature-Based Approach for Predicting Conotoxin Superfamily. Protein Pept. Lett..

[18] Yin J.B., Fan Y.X., Shen H.B. (2011). Conotoxin Superfamily Prediction Using Diffusion Maps Dimensionality Reduction and Subspace Classifier. Curr. Protein Pept. Sci..

[19] Zaki N., Wolfsheimer S., Nuel G., Khuri S. (2011). Conotoxin protein classification using free scores of words and support vector machines. BMC Bioinform..

[20] Koua D., Laht S., Kaplinski L., Stöcklin R., Remm M., Favreau P., Lisacek F. (2013). Position-specific scoring matrix and hidden markov model complement each other for the prediction of conopeptide superfamilies. Biochim. Et Biophys. Acta BBA Proteins Proteom..

[21] Lavergne V., Dutertre S., Jin A.-h., Lewis R.J., Taft R.J., Alewood P.F. (2013). Systematic interrogation of the *Conus marmoreus* venom duct transcriptome with ConoSorter reveals 158 novel conotoxins and 13 new gene superfamilies. BMC Genom..

[22] Koua D., Ebou A., Dutertre S. (2021). Improved prediction of conopeptide superfamilies with ConoDictor 2.0. Bioinform. Adv..

[23] Li Q., Watkins M., Robinson S.D., Safavi-Hemami H., Yandell M. (2018). Discovery of Novel Conotoxin Candidates Using Machine Learning. Toxins.

[24] Monroe L.K., Truong D.P., Miner J.C., Adikari S.H., Sasiene Z.J., Fenimore P.W., Alexandrov B., Williams R.F., Nguyen H.B. (2023). Conotoxin Prediction: New Features to Increase Prediction Accuracy. Toxins.

[25] Wang Y., Zhai Y., Ding Y., Zou Q. (2024). SBSM-Pro: Support bio-sequence machine for proteins. Sci. China Inf. Sci..

[26] Liang C., Wang D., Zhang H., Zhang S., Guo F. (2024). Robust Tensor Subspace Learning for Incomplete Multi-View Clustering. IEEE Trans. Knowl. Data Eng..

[27] Pang C., Qiao J., Zeng X., Zou Q., Wei L. (2023). Deep generative models in de novo drug molecule generation. J. Chem. Inf. Model..

[28] Kaas Q., Yu R., Jin A.H., Dutertre S., Craik D.J. (2011). ConoServer: Updated content, knowledge, and discovery tools in the conopeptide database. Nucleic Acids Res..

[29] Kaas Q., Westermann J.C., Halai R., Wang C.K.L., Craik D.J. (2008). ConoServer, a database for conopeptide sequences and structures. Bioinformatics.

[30] Kaas Q., Westermann J.-C., Craik D.J. (2010). Conopeptide characterization and classifications: An analysis using ConoServer. Toxicon.

[31] Liu M., Li C., Chen R., Cao D., Zeng X. (2023). Geometric Deep Learning for Drug Discovery. Expert Syst. Appl..

[32] Liu T., Huang J., Luo D., Ren L., Ning L., Huang J., Lin H., Zhang Y. (2024). Cm-siRPred: Predicting chemically modified siRNA efficiency based on multi-view learning strategy. Int. J. Biol. Macromol..

[33] Wang R., Jiang Y., Jin J., Yin C., Yu H., Wang F., Feng J., Su R., Nakai K., Zou Q. (2023). DeepBIO: An automated and interpretable deep-learning platform for high-throughput biological sequence prediction, functional annotation and visualization analysis. Nucleic Acids Res..

[34] Tan J.X., Li S.H., Zhang Z.M., Chen C.X., Chen W., Tang H., Lin H. (2019). Identification of hormone binding proteins based on machine learning methods. Math. Biosci. Eng..

[35] Zhang H.Q., Liu S.H., Li R., Yu J.W., Ye D.X., Yuan S.S., Lin H., Huang C.-B., Tang H. (2024). MIBPred: Ensemble Learning-Based Metal Ion-Binding Protein Classifier. ACS Omega.

[36] Wei L., He W., Malik A., Su R., Cui L., Manavalan B. (2020). Computational prediction and interpretation of cell-specific replication origin sites from multiple eukaryotes by exploiting stacking framework. Brief. Bioinform..

[37] Xu Y., Liu T., Yang Y., Kang J., Ren L., Ding H., Zhang Y. (2024). ACVPred: Enhanced prediction of anti-coronavirus peptides by transfer learning combined with data augmentation. Future Gener. Comput. Syst..

[38] Liu T., Qiao H., Wang Z., Yang X., Pan X., Yang Y., Ye X., Sakurai T., Lin H., Zhang Y. (2024). CodLncScape Provides a Self-Enriching Framework for the Systematic Collection and Exploration of Coding LncRNAs. Adv. Sci..

[39] Sun Z. (2020). ICTC-RAAC: An improved web predictor for identifying the types of ion channel-targeted conotoxins by using reduced amino acid cluster descriptors *Comput*. Biol. Chem..

[40] Truong D.P., Monroe L.K., Williams R.F., Nguyen H.B. (2024). Machine learning framework for conotoxin class and molecular target prediction. Toxins.

[41] Woodward S.R., Cruz L.J., Olivera B.M., Hillyard D.R. (1990). Constant and hypervariable regions in conotoxin propeptides. EMBO J..

[42] Akondi K.B., Muttenthaler M., Dutertre S., Kaas Q., Craik D.J., Lewis R.J., Alewood P.F. (2014). Discovery, synthesis and development of structure-activity relationships of conotoxins. Chem. Rev..

[43] Robinson S.D., Safavi-Hemami H., McIntosh L.D., Purcell A.W., Norton R.S., Papenfuss A.T. (2014). Diversity of conotoxin gene superfamilies in the venomous snail, *Conus victoriae*. PLoS ONE.

[44] Jin A.H., Dekan Z., Smout M.J., Wilson D., Dutertre S., Vetter I., Lewis R.J., Loukas A., Daly N.L., Alewood P.F. (2017). Conotoxin Φ-MiXXVIIA from the superfamily G2 employs a novel cysteine framework that mimics granulin and displays anti-apoptotic activity. Angew. Chem. Int. Ed..

[45] Chang D., Duda T.F. (2012). Extensive and continuous duplication facilitates rapid evolution and diversification of gene families. Mol. Biol. Evol..

[46] Conticello S.G., Gilad Y., Avidan N., Ben-Asher E., Levy Z., Fainzilber M. (2001). Mechanisms for evolving hypervariability: The case of conopeptides. Mol. Biol. Evol..

[47] Kumar P.S., Kumar D.S., Umamaheswari S. (2015). A perspective on toxicology of *Conus* venom peptides. Asian Pac. J. Trop. Med..

[48] Zhang Y., Pan X., Shi T., Gu Z., Yang Z., Liu M., Xu Y., Yang Y., Ren L., Song X. (2024). P450Rdb: A manually curated database of reactions catalyzed by cytochrome P450 enzymes. J. Adv. Res..

[49] Franco A., Pisarewicz K., Moller C., Mora D., Fields G.B., Marí F., Cimino G., Gavagnin M. (2006). Hyperhydroxylation: A new strategy for neuronal targeting by venomous marine molluscs. Molluscs: From Chemo-Ecological Study to Biotechnological Application.

[50] Ho T.N.T., Lee H.S., Swaminathan S., Goodwin L., Rai N., Ushay B., Lewis R.J., Rosengren K.J., Conibear A.C. (2021). Posttranslational modifications of α-conotoxins: Sulfotyrosine and C-terminal amidation stabilise structures and increase acetylcholine receptor binding. RSC Med. Chem..

[51] Wang L., Liu J., Ren Z., Chen Y., Xu A. (2017). Discovery of two P-superfamily conotoxins, lt9a and lt9b, with different modifications on voltage-sensitive sodium channels. Toxicon.

[52] Ao C., Jiao S., Wang Y., Yu L., Zou Q. (2022). Biological Sequence Classification: A Review on Data and General Methods. Research.

[53] Lewis R.J., Dutertre S., Vetter I., Christie M.J. (2012). *Conus* venom peptide pharmacology. Pharmacol. Rev..

[54] Mir R., Karim S., Kamal M.A., Wilson C.M., Mirza Z. (2016). Conotoxins: Structure, therapeutic potential and pharmacological applications. Curr. Pharm. Des..

[55] Robinson S.D., Safavi-Hemami H. (2017). Venom peptides as pharmacological tools and therapeutics for diabetes. Neuropharmacology.

[56] Liao Y., Peng C., Zhu Y., Fu J., Ruan Z., Shi Q., Gao B. (2022). High conopeptide diversity in *Conus striatus*: Revealed by integration of two transcriptome sequencing platforms. Front. Mar. Sci..

[57] Peng C., Yao G., Gao B.-M., Fan C.-X., Bian C., Wang J., Cao Y., Wen B., Zhu Y., Ruan Z. (2016). High-throughput identification of novel conotoxins from the Chinese tubular cone snail (*Conus betulinus*) by multi-transcriptome sequencing. GigaScience.

[58] Li X., Liu H., Gao C., Li Y., Jia D., Yang Y., Yang J., Wei Z., Jiang T., Yu R. (2020). ConoMode, a database for conopeptide binding modes. Database: J. Biol. Databases Curation.

[59] Younis S., Rashid S. (2017). Alpha conotoxin-BuIA globular isomer is a competitive antagonist for oleoyl-L-alpha-lysophosphatidic acid binding to LPAR6; a molecular dynamics study. PLoS ONE.

[60] Cordeiro S., Finol-Urdaneta R.K., Köpfer D., Markushina A., Song J., French R.J., Kopec W., de Groot B.L., Giacobassi M.J., Leavitt L.S. (2019). Conotoxin κM-RIIIJ, a tool targeting asymmetric heteromeric Kv1 channels. Proc. Natl. Acad. Sci. USA.

[61] Kohn A.J., Saunders P.R., Wiener S. (1960). Preliminary studies on the venom of the marine snail *Conus*. Ann. N. Y. Acad. Sci..

[62] Jin H., Cui D., Fan Y., Li G., Zhong Z., Wang Y. (2024). Recent advances in bioaffinity strategies for preclinical and clinical drug discovery: Screening natural products, small molecules and antibodies. Drug Discov. Today.

[63] Chen S., Shen C., Li W., Fan Y., Yang D.-H., Wang Y., Feng R., Li G., Zhong Z. (2024). Recent advances in bioactivity-guided drug screening strategies for pre-clinical and clinical drug discovery. TrAC Trends Anal. Chem..

[64] Hu H., Bandyopadhyay P.K., Olivera B.M., Yandell M. (2011). Characterization of the *Conus bullatus* genome and its venom-duct transcriptome. BMC Genom..

[65] Hu H., Bandyopadhyay P.K., Olivera B.M., Yandell M. (2012). Elucidation of the molecular envenomation strategy of the cone snail *Conus geographus* through transcriptome sequencing of its venom duct. BMC Genom..

[66] Terrat Y., Biass D., Dutertre S., Favreau P., Remm M., Stöcklin R., Piquemal D., Ducancel F. (2012). High-resolution picture of a venom gland transcriptome: Case study with the marine snail *Conus consors*. Toxicon.

[67] Hackney C.M., Salcedo P.F., Mueller E., Koch T.L., Kjelgaard L.D., Watkins M., Zachariassen L.G., Tuelung P.S., McArthur J.R., Adams D.J. (2023). A previously unrecognized superfamily of macro-conotoxins includes an inhibitor of the sensory neuron calcium channel Cav2.3. PLOS Biol..

[68] Nguyen L.T.T., Craik D.J., Kaas Q. (2023). Bibliometric review of the literature on cone snail peptide toxins from 2000 to 2022. Mar. Drugs.

[69] Espiritu M.J., Taylor J.K., Sugai C.K., Thapa P., Loening N.M., Gusman E., Baoanan Z.G., Baumann M.H., Bingham J.-P. (2023). Characterization of the native disulfide isomers of the novel χ-conotoxin PnID: Implications for further increasing conotoxin diversity. Mar. Drugs.

[70] Ebou A., Koua D., Addablah A., Kakou-Ngazoa S., Dutertre S. (2021). Combined proteotranscriptomic-based strategy to discover novel antimicrobial peptides from cone snails. Biomedicines.

[71] Lu A., Watkins M., Li Q., Robinson S.D., Concepcion G.P., Yandell M., Weng Z., Olivera B.M., Safavi-Hemami H., Fedosov A.E. (2020). Transcriptomic profiling reveals extraordinary diversity of venom peptides in unexplored predatory gastropods of the genus clavus. Genome Biol. Evol..

[72] Hu S.H., Gehrmann J., Alewood P.F., Craik D.J., Martin J.L. (1997). Crystal structure at 1.1 Å resolution of α-conotoxin PnIB:  Comparison with α-conotoxins PnIA and GI. Biochemistry.

[73] Hill J.M., Alewood P.F., Craik D.J. (2000). Conotoxin TVIIA, a novel peptide from the venom of *Conus tulipa*. Eur. J. Biochem..

[74] Wilson D.T., Bansal P.S., Carter D.A., Vetter I., Nicke A., Dutertre S., Daly N.L. (2020). Characterisation of a novel a-superfamily conotoxin. Biomedicines.

[75] Kang T.S., Jois S.D.S., Kini R.M. (2006). Solution structures of two structural isoforms of CMrVIA χ/λ-conotoxin. Biomacromolecules.

[76] Altschul S.F., Gish W., Miller W., Myers E.W., Lipman D.J. (1990). Basic local alignment search tool. J. Mol. Biol..

[77] Eddy S.R. (1998). Profile hidden markov models. Bioinformatics.

[78] Zamora-Bustillos R., Martínez-Núñez M.A., Aguilar M.B., Collí-Dula R.C., Brito-Domínguez D.A. (2021). Identification of novel conotoxin precursors from the cone snail *Conus spurius* by high-throughput RNA sequencing. Mar. Drugs.

[79] Dao F.Y., Yang H., Su Z.D., Yang W., Wu Y., Ding H., Chen W., Tang H., Lin H. (2017). Recent Advances in Conotoxin Classification by Using Machine Learning Methods. Molecules.

[80] Robinson S.D., Norton R.S. (2014). Conotoxin gene superfamilies. Mar. Drugs.

[81] Lee S.F., Davey L. (2017). Disulfide bonds: A key modification in bacterial extracytoplasmic proteins. J. Dent. Res..

[82] Fu J., Gao J., Liang Z., Yang D. (2021). PDI-regulated disulfide bond formation in protein folding and biomolecular assembly. Molecules.

[83] Turner A., Kaas Q., Craik D.J. (2020). Hormone-like conopeptides—New tools for pharmaceutical design. RSC Med. Chem..

[84] Biggs J.S., Watkins M., Puillandre N., Ownby J.-P., Lopez-Vera E., Christensen S., Moreno K.J., Bernaldez J., Licea-Navarro A., Corneli P.S. (2010). Evolution of *Conus* peptide toxins: Analysis of *Conus californicus* reeve, 1844. Mol. Phylogenetics Evol..

[85] Elliger C.A., Richmond T.A., Lebaric Z.N., Pierce N.T., Sweedler J.V., Gilly W.F. (2011). Diversity of conotoxin types from *Conus californicus* reflects a diversity of prey types and a novel evolutionary history. Toxicon.

[86] Balsara R., Li N., Weber-Adrian D., Huang L., Castellino F.J. (2012). Opposing action of conantokin-G on synaptically and extrasynaptically-activated NMDA receptors. Neuropharmacology.

[87] Malmberg A.B., Gilbert H., McCabe T.R., Basbaum A.I. (2003). Powerful antinociceptive effects of the cone snail venom-derived subtype-selective NMDA receptor antagonists conantokins G and T. Pain.

[88] Hama A., Sagen J. (2009). Antinociceptive effects of the marine snail peptides conantokin-G and conotoxin MVIIA alone and in combination in rat models of pain. Neuropharmacology.

[89] Balsara R., Dang A., Donahue D.L., Snow T., Castellino F.J. (2015). Conantokin-G attenuates detrimental effects of NMDAR hyperactivity in an ischemic rat model of stroke. PLoS ONE.

[90] Jacob R.B., McDougal O.M. (2010). The M-superfamily of conotoxins: A review. Cell. Mol. Life Sci..

[91] Zhou M., Wang L., Wu Y., Zhu X., Feng Y., Chen Z., Li Y., Sun D., Ren Z., Xu A. (2013). Characterizing the evolution and functions of the M-superfamily conotoxins. Toxicon.

[92] Ho T.N., Tran T.H., Le H.S., Lewis R.J. (2025). Advances in the synthesis and engineering of conotoxins. Eur. J. Med. Chem..

[93] Gao B., Peng C., Yang J., Yi Y., Zhang J., Shi Q. (2017). Cone snails: A big store of conotoxins for novel drug discovery. Toxins.

[94] Halai R., Craik D.J. (2009). Conotoxins: Natural product drug leads. Nat. Prod. Rep..

[95] Green B.R., Bulaj G., Norton R.S. (2014). Structure and function of μ-conotoxins, peptide-based sodium channel blockers with analgesic activity. Future Med. Chem..

[96] Ekberg J., Craik D.J., Adams D.J. (2008). Conotoxin modulation of voltage-gated sodium channels. Int. J. Biochem. Cell Biol..

[97] Ramírez D., Gonzalez W., Fissore R.A., Carvacho I. (2017). Conotoxins as tools to understand the physiological function of voltage-gated calcium (CaV) channels. Mar. Drugs.

[98] Pope J.E., Deer T.R. (2013). Ziconotide: A clinical update and pharmacologic review. Expert Opin. Pharmacother..

[99] Safavi-Hemami H., Brogan S.E., Olivera B.M. (2019). Pain therapeutics from cone snail venoms: From ziconotide to novel non-opioid pathways. J. Proteom..

[100] Miljanich G.P. (2004). Ziconotide: Neuronal calcium channel blocker for treating severe chronic pain. Curr. Med. Chem..

[101] Ferber M., Sporning A., Jeserich G., DeLaCruz R., Watkins M., Olivera B.M., Terlau H. (2003). A novel *Conus* peptide ligand for K+channels*. J. Biol. Chem..

[102] Ferber M., Al-Sabi A., Stocker M., Olivera B.M., Terlau H. (2004). Identification of a mammalian target of κM-conotoxin RIIIK. Toxicon.

[103] Hone A.J., McIntosh J.M. (2023). Nicotinic acetylcholine receptors: Therapeutic targets for novel ligands to treat pain and inflammation. Pharmacol. Res..

[104] Sadeghi M., McArthur J.R., Finol-Urdaneta R.K., Adams D.J. (2017). Analgesic conopeptides targeting G protein-coupled receptors reduce excitability of sensory neurons. Neuropharmacology.

[105] Craig A.G., Norberg T., Griffin D., Hoeger C., Akhtar M., Schmidt K., Low W., Dykert J., Richelson E., Navarro V. (1999). Contulakin-G, an O-glycosylated invertebrate neurotensin*. J. Biol. Chem..

[106] Pei S., Wang N., Mei Z., Zhangsun D., Craik D.J., McIntosh J.M., Zhu X., Luo S. (2024). Conotoxins targeting voltage-gated sodium ion channels. Pharmacol. Rev..

[107] Olivera B.M., Cruz L.J., De Santos V., LeCheminant G., Griffin D., Zeikus R., McIntosh J.M., Galyean R., Varga J. (1987). Neuronal calcium channel antagonists. Discrimination between calcium channel subtypes using.omega.-conotoxin from *Conus magus* venom. Biochemistry.

[108] Brady R.M., Zhang M., Gable R., Norton R.S., Baell J.B. (2013). De novo design and synthesis of a μ-conotoxin KIIIA peptidomimetic. Bioorganic Med. Chem. Lett..

[109] Duda T.F., Palumbi S.R. (1999). Molecular genetics of ecological diversification: Duplication and rapid evolution of toxin genes of the venomous gastropod *Conus*. Proc. Natl. Acad. Sci. USA.

[110] Wang H., Li X., Zhangsun D., Yu G., Su R., Luo S. (2019). The α9α10 nicotinic acetylcholine receptor antagonist αO-conotoxin GeXIVA [1,2] alleviates and reverses chemotherapy-induced neuropathic pain. Mar. Drugs.

[111] Romero H.K., Christensen S.B., Di Cesare Mannelli L., Gajewiak J., Ramachandra R., Elmslie K.S., Vetter D.E., Ghelardini C., Iadonato S.P., Mercado J.L. (2017). Inhibition of α9α10 nicotinic acetylcholine receptors prevents chemotherapy-induced neuropathic pain. Proc. Natl. Acad. Sci. USA.

[112] Zouridakis M., Papakyriakou A., Ivanov I.A., Kasheverov I.E., Tsetlin V., Tzartos S., Giastas P. (2019). Crystal structure of the monomeric extracellular domain of α9 nicotinic receptor subunit in complex with α-conotoxin RgIA: Molecular dynamics insights into RgIA binding to α9α10 nicotinic receptors. Front. Pharmacol..

[113] Smallwood T.B., Clark R.J. (2021). Advances in venom peptide drug discovery: Where are we at and where are we heading?. Expert Opin. Drug Discov..

[114] Safavi-Hemami H., Gajewiak J., Karanth S., Robinson S.D., Ueberheide B., Douglass A.D., Schlegel A., Imperial J.S., Watkins M., Bandyopadhyay P.K. (2015). Specialized insulin is used for chemical warfare by fish-hunting cone snails. Proc. Natl. Acad. Sci..

[115] Robinson S.D., Safavi-Hemami H. (2016). Insulin as a weapon. Toxicon.

[116] Bayrhuber M., Vijayan V., Ferber M., Graf R., Korukottu J., Imperial J., Garrett J.E., Olivera B.M., Terlau H., Zweckstetter M. (2005). Conkunitzin-S1 is the first member of a new kunitz-type neurotoxin family: Structural and Functional Characterization*. J. Biol. Chem..

[117] Finol-Urdaneta R.K., Remedi M.S., Raasch W., Becker S., Clark R.B., Strüver N., Pavlov E., Nichols C.G., French R.J., Terlau H. (2012). Block of Kv1.7 potassium currents increases glucose-stimulated insulin secretion. EMBO Mol. Med..

[118] Hemu X., Tam J.P. (2017). Macrocyclic antimicrobial peptides engineered from ω-conotoxin. Curr. Pharm. Des..

[119] Wang M., Liao Z., Zhangsun D., Wu Y., Luo S. (2024). Engineering enhanced antimicrobial properties in α-conotoxin RgIA through D-type amino acid substitution and incorporation of lysine and leucine residues. Molecules.

[120] Mei D., Lin Z., Fu J., He B., Gao W., Ma L., Dai W., Zhang H., Wang X., Wang J. (2015). The use of α-conotoxin ImI to actualize the targeted delivery of paclitaxel micelles to α7 nAChR-overexpressing breast cancer. Biomaterials.

[121] Mei D., Zhao L., Chen B., Zhang X., Wang X., Yu Z., Ni X., Zhang Q. (2018). α-conotoxin ImI-modified polymeric micelles as potential nanocarriers for targeted docetaxel delivery to α7-nAChR overexpressed non-small cell lung cancer. Drug Deliv..

[122] Osipov A.V., Terpinskaya T.I., Yanchanka T., Balashevich T., Zhmak M.N., Tsetlin V.I., Utkin Y.N. (2020). α-conotoxins enhance both the in vivo suppression of ehrlich carcinoma growth and in vitro reduction in cell viability elicited by cyclooxygenase and lipoxygenase inhibitors. Mar. Drugs.

[123] Mondal S., Bhavna R., Mohan Babu R., Ramakumar S. (2006). Pseudo amino acid composition and multi-class support vector machines approach for conotoxin superfamily classification. J. Theor. Biol..

[124] Zaki N., Sibai F., Campbell P. (2011). Conotoxin protein classification using pairwise comparison and amino acid composition: Toxin-aam. Proceedings of the 13th Annual Conference on Genetic and Evolutionary Computation.

[125] Yuan L.-F., Ding C., Guo S.-H., Ding H., Chen W., Lin H. (2013). Prediction of the types of ion channel-targeted conotoxins based on radial basis function network. Toxicol. Vitr..

[126] Ding H., Deng E.-Z., Yuan L.-F., Liu L., Lin H., Chen W., Chou K.-C. (2014). iCTX-Type: A Sequence-Based Predictor for Identifying the Types of Conotoxins in Targeting Ion Channels. BioMed Res. Int..

[127] Wu Y., Zheng Y., Tang H. (2016). Identifying the Types of Ion Channel-Targeted Conotoxins by Incorporating New Properties of Residues into Pseudo Amino Acid Composition. BioMed Res. Int..

[128] Zhang L., Zhang C., Gao R., Yang R., Song Q. (2016). Using the SMOTE technique and hybrid features to predict the types of ion channel-targeted conotoxins. J. Theor. Biol..

[129] Hofmann K. (2000). Sensitive protein comparisons with profiles and hidden markov models. Brief. Bioinform..

[130] Nastou K.C., Tsaousis G.N., Papandreou N.C., Hamodrakas S.J. (2016). MBPpred: Proteome-wide detection of membrane lipid-binding proteins using profile hidden markov models. Biochim. Et Biophys. Acta BBA Proteins Proteom..

[131] Huo L., Zhang H., Huo X., Yang Y., Li X., Yin Y. (2017). pHMM-tree: Phylogeny of profile hidden markov models. Bioinformatics.

[132] Feldbauer R., Gosch L., Lüftinger L., Hyden P., Flexer A., Rattei T. (2021). DeepNOG: Fast and accurate protein orthologous group assignment. Bioinformatics.

[133] Koua D., Brauer A., Laht S., Kaplinski L., Favreau P., Remm M. (2012). ConoDictor: A tool for prediction of conopeptide superfamilies. Nucleic Acids Res..

[134] Waris M., Ahmad K., Kabir M., Hayat M. (2016). Identification of DNA binding proteins using evolutionary profiles position specific scoring matrix. Neurocomputing.

[135] Liu X., Lu Y., Wang L., Geng W., Shi X., Zhang X. (2023). RF-PSSM: A combination of rotation forest algorithm and position-specific scoring matrix for improved prediction of protein-protein interactions between hepatitis C virus and human. Big Data Min. Anal..

[136] Ruan X., Xia S., Li S., Su Z., Yang J. (2024). Hybrid framework for membrane protein type prediction based on the PSSM. Sci. Rep..

[137] Manavalan B., Lee J. (2022). FRTpred: A novel approach for accurate prediction of protein folding rate and type. Comput. Biol. Med..

[138] Nithiyanandam S., Sangaraju V.K., Manavalan B., Lee G. (2023). Computational prediction of protein folding rate using structural parameters and network centrality measures. Comput. Biol. Med..

[139] Gowd K.H., Dewan K.K., Iengar P., Krishnan K.S., Balaram P. (2008). Probing peptide libraries from *Conus achatinus* using mass spectrometry and cDNA sequencing: Identification of δ and ω-conotoxins. J. Mass. Spectrom..

[140] Wang X.F., Wang J.M., Wang X.L., Zhang Y. (2017). Predicting the Types of Ion Channel-Targeted Conotoxins Based on AVC-SVM Model. BioMed Res. Int..

[141] Zuo Y., Li Y., Chen Y., Li G., Yan Z., Yang L. (2017). PseKRAAC: A flexible web server for generating pseudo K-tuple reduced amino acids composition. Bioinformatics.

[142] Yang S., Liu D., Song Y., Liang Y., Yu H., Zuo Y. (2024). Designing a structure-function alphabet of helix based on reduced amino acid clusters. Arch. Biochem. Biophys..

[143] Zheng L., Liu D., Li Y.A., Yang S., Liang Y., Xing Y., Zuo Y. (2022). RaacFold: A webserver for 3D visualization and analysis of protein structure by using reduced amino acid alphabets. Nucleic Acids Res..

[144] Liang Y., Yang S., Zheng L., Wang H., Zhou J., Huang S., Yang L., Zuo Y. (2022). Research progress of reduced amino acid alphabets in protein analysis and prediction. Comput. Struct. Biotechnol. J..

[145] Zheng L., Liu D., Yang W., Yang L., Zuo Y. (2021). RaacLogo: A new sequence logo generator by using reduced amino acid clusters. Brief. Bioinform..

[146] Cortes C., Vapnik V. (1995). Support-vector networks. Mach. Learn..

[147] Guo Y., Zhang Z., Tang F. (2021). Feature selection with kernelized multi-class support vector machine. Pattern Recognit..

[148] Liu Q., Chen C., Zhang Y., Hu Z. (2011). Feature selection for support vector machines with RBF kernel. Artif. Intell. Rev..

[149] Madugula S.S., Pujar P., Nammi B., Wang S., Jayasinghe-Arachchige V.M., Pham T., Mashburn D., Artiles M., Liu J. (2024). Identification of family-specific features in Cas9 and Cas12 proteins: A machine learning approach using complete protein feature spectrum. J. Chem. Inf. Model..

[150] Rahman M.S., Rahman M.K., Kaykobad M., Rahman M.S. (2018). isGPT: An optimized model to identify sub-golgi protein types using SVM and random forest based feature selection. Artif. Intell. Med..

[151] Liu S., Shi T., Yu J., Li R., Lin H., Deng K. (2024). Research on Bitter Peptides in the Field of Bioinformatics: A Comprehensive Review. Int. J. Mol. Sci..

[152] Liu B., Gao X., Zhang H. (2019). BioSeq-Analysis2.0: An updated platform for analyzing DNA, RNA and protein sequences at sequence level and residue level based on machine learning approaches. Nucleic Acids Res..

[153] Li H., Pang Y., Liu B. (2021). BioSeq-BLM: A platform for analyzing DNA, RNA, and protein sequences based on biological language models. Nucleic Acids Res..

[154] Wei Z.S., Han K., Yang J.Y., Shen H.B., Yu D.J. (2016). Protein–protein interaction sites prediction by ensembling SVM and sample-weighted random forests. Neurocomputing.

[155] Jia S., Hu X., Sun L. (2013). The comparison between random forest and support vector machine algorithm for predicting β-hairpin motifs in proteins. Engineering.

[156] Chen X., Wang C.C., Yin J., You Z.H. (2018). Novel human miRNA-disease association inference based on random forest. Mol. Ther. Nucleic Acids.

[157] Wu Q.W., Xia J.F., Ni J.C., Zheng C.H. (2021). GAERF: Predicting lncRNA-disease associations by graph auto-encoder and random forest. Brief. Bioinform..

[158] Aybey B., Zhao S., Brors B., Staub E. (2023). Immune cell type signature discovery and random forest classification for analysis of single cell gene expression datasets. Front. Immunol..

[159] Puillandre N., Duda T.F., Meyer C., Olivera B.M., Bouchet P. (2014). One, four or 100 genera? A new classification of the cone snails. J. Molluscan Stud..

[160] Naamati G., Askenazi M., Linial M. (2009). ClanTox: A classifier of short animal toxins. Nucleic Acids Res..

[161] Gupta S., Kapoor P., Chaudhary K., Gautam A., Kumar R., Raghava G.P.S. (2013). In silico approach for predicting toxicity of peptides and proteins. PLoS ONE.

[162] Cole T.J., Brewer M.S. (2019). TOXIFY: A deep learning approach to classify animal venom proteins. PeerJ.

[163] Wheeler T.J., Eddy S.R. (2013). nhmmer: DNA homology search with profile HMMs. Bioinformatics.

[164] Conticello S.G., Kowalsman N.D., Jacobsen C., Yudkovsky G., Sato K., Elazar Z., Petersen C.M., Aronheim A., Fainzilber M. (2003). The prodomain of a secreted hydrophobic mini-protein facilitates its export from the endoplasmic reticulum by hitchhiking on sorting receptors. J. Biol. Chem..

[165] Buczek O., Olivera B.M., Bulaj G. (2004). Propeptide does not act as an intramolecular chaperone but facilitates protein disulfide isomerase-assisted folding of a conotoxin precursor. Biochemistry.

[166] Yu H.F., Huang F.L., Lin C.J. (2011). Dual coordinate descent methods for logistic regression and maximum entropy models. Mach. Learn..

[167] Heerdt G., Zanotto L., Souza P.C.T., Araujo G., Skaf M.S. (2020). Collision cross section calculations using HPCCS. Methods Mol. Biol..

[168] Chi Q.N., Jia S.X., Yin H., Wang L.E., Fu X.Y., Ma Y.N., Sun M.P., Qi Y.K., Li Z.B., Du S.S. (2023). Efficient synthesis and anticancer evaluation of spider toxin peptide LVTX-8-based analogues with enhanced stability. Bioorganic Chem..

[169] Greco I., Molchanova N., Holmedal E., Jenssen H., Hummel B.D., Watts J.L., Håkansson J., Hansen P.R., Svenson J. (2020). Correlation between hemolytic activity, cytotoxicity and systemic in vivo toxicity of synthetic antimicrobial peptides. Sci. Rep..

[170] Robinson S.D., Undheim E.A.B., Ueberheide B., King G.F. (2017). Venom peptides as therapeutics: Advances, challenges and the future of venom-peptide discovery. Expert Rev. Proteom..

[171] (2024). Spotlight on researchers during a wet-lab to dry-lab transition period: An interview with guifen liu and qi wang. Commun. Biol..

[172] Asamitsu S., Yabuki Y., Matsuo K., Kawasaki M., Hirose Y., Kashiwazaki G., Chandran A., Bando T., Wang D.O., Sugiyama H. (2023). RNA G-quadruplex organizes stress granule assembly through DNAPTP6 in neurons. Sci. Adv..

[173] Madani A., Krause B., Greene E.R., Subramanian S., Mohr B.P., Holton J.M., Olmos J.L., Xiong C., Sun Z.Z., Socher R. (2023). Large language models generate functional protein sequences across diverse families. Nat. Biotechnol..

[174] Ferruz N., Schmidt S., Höcker B. (2022). ProtGPT2 is a deep unsupervised language model for protein design. Nat. Commun..

[175] Sumida K.H., Núñez-Franco R., Kalvet I., Pellock S.J., Wicky B.I.M., Milles L.F., Dauparas J., Wang J., Kipnis Y., Jameson N. (2024). Improving protein expression, stability, and function with ProteinMPNN. J. Am. Chem. Soc..

[176] Guo M., Li Z., Deng X., Luo D., Yang J., Xue W. (2024). ConoDL: A Deep Learning Framework for Rapid Generation and Prediction of Conotoxins. J. Comput. Mol. Des..

[177] Tay Y., Dehghani M., Bahri D., Metzler D. (2022). Efficient transformers: A survey. ACM Comput. Surv..

[178] Vaswani A., Shazeer N., Parmar N., Uszkoreit J., Jones L., Gomez A.N., Kaiser Ł.U., Polosukhin I. (2017). Attention is all you need. Advances in Neural Information Processing Systems.

[179] Yang L., Yang G., Bing Z., Tian Y., Huang L., Niu Y., Yang L. (2022). Accelerating the discovery of anticancer peptides targeting lung and breast cancers with the wasserstein autoencoder model and PSO algorithm. Brief. Bioinform..

[180] Cao Q., Ge C., Wang X., Harvey P.J., Zhang Z., Ma Y., Wang X., Jia X., Mobli M., Craik D.J. (2023). Designing antimicrobial peptides using deep learning and molecular dynamic simulations. Brief. Bioinform..

[181] Das P., Sercu T., Wadhawan K., Padhi I., Gehrmann S., Cipcigan F., Chenthamarakshan V., Strobelt H., dos Santos C., Chen P.Y. (2021). Accelerated antimicrobial discovery via deep generative models and molecular dynamics simulations. Nat. Biomed. Eng..

[182] Ali F., Khalid M., Almuhaimeed A., Masmoudi A., Alghamdi W., Yafoz A. (2024). IP-GCN: A deep learning model for prediction of insulin using graph convolutional network for diabetes drug design. J. Comput. Sci..

[183] Ali F., Khalid M., Masmoudi A., Alghamdi W., Yafoz A., Alsini R. (2024). VEGF-ERCNN: A Deep Learning-based Model for Prediction of Vascular Endothelial Growth Factor using Ensemble Residual CNN. J. Comput. Sci..

[184] Ali F., Almuhaimeed A., Khalid M., Alshanbari H., Masmoudi A., Alsini R.J.M. (2024). DEEP-EP: Identification of epigenetic protein by ensemble residual convolutional neural network for drug discovery. Methods.

[185] Alsini R., Almuhaimeed A., Ali F., Khalid M., Farrash M., Masmoudi A. (2024). Deep-VEGF: Deep stacked ensemble model for prediction of vascular endothelial growth factor by concatenating gated recurrent unit with two-dimensional convolutional neural network. J. Biomol. Struct. Dyn..

[186] Sohl-Dickstein J., Weiss E.A., Maheswaranathan N., Ganguli S. (2015). Deep unsupervised learning using nonequilibrium thermodynamics. arXiv.

[187] Cai Z., Xiong Z., Xu H., Wang P., Li W., Pan Y. (2021). Generative adversarial networks: A survey toward private and secure applications. ACM Comput. Surv..

[188] Sohn K., Yan X., Lee H., Cortes C., Lawrence N.D., Lee D.D., Sugiyama M., Garnett R. (2015). Learning structured output representation using deep conditional generative models. Advances in Neural Information Processing Systems 28 (Nips 2015).

[189] Lu J., Batra D., Parikh D., Lee S., Wallach H., Larochelle H., Beygelzimer A., d’Alche-Buc F., Fox E., Garnett R. (2019). ViLBERT: Pretraining task-agnostic visiolinguistic representations for vision-and-language tasks. Advances in Neural Information Processing Systems 32 (Nips 2019).

[190] Scarselli F., Gori M., Tsoi A.C., Hagenbuchner M., Monfardini G. (2009). The graph neural network model. IEEE Trans. Neural Netw..

[191] Gao Y., Chuai G., Yu W., Qu S., Liu Q. (2020). Data imbalance in CRISPR off-target prediction. Brief. Bioinform..

[192] Tang X., Cai L., Meng Y., Gu C., Yang J., Yang J. (2021). A novel hybrid feature selection and ensemble learning framework for unbalanced cancer data diagnosis with transcriptome and functional proteomic. IEEE Access.

[193] Pham N.T., Phan L.T., Seo J., Kim Y., Song M., Lee S., Jeon Y.J., Manavalan B. (2023). Advancing the accuracy of SARS-CoV-2 phosphorylation site detection via meta-learning approach. Brief. Bioinform..

[194] Pham N.T., Zhang Y., Rakkiyappan R., Manavalan B. (2024). HOTGpred: Enhancing human O-linked threonine glycosylation prediction using integrated pretrained protein language model-based features and multi-stage feature selection approach. Comput. Biol. Med..

[195] Joshi M., Singh B.K. (2024). Deep Learning Techniques for Brain Lesion Classification Using Various MRI (from 2010 to 2022): Review and Challenges. Medinformatics.

[196] Zulfiqar H., Guo Z., Ahmad R.M., Ahmed Z., Cai P., Chen X., Zhang Y., Lin H., Shi Z. (2024). Deep-STP: A deep learning-based approach to predict snake toxin proteins by using word embeddings. Front. Med..

[197] Wang H., Li J., Wu H., Hovy E., Sun Y. (2023). Pre-trained language models and their applications. Engineering.

[198] Zhuang F., Qi Z., Duan K., Xi D., Zhu Y., Zhu H., Xiong H., He Q. (2021). A comprehensive survey on transfer learning. Proc. IEEE.

[199] Weiss K., Khoshgoftaar T.M., Wang D. (2016). A survey of transfer learning. J. Big Data.

[200] Charuvaka A., Rangwala H. (2014). Classifying protein sequences using regularized multi-task learning. IEEE/ACM Trans. Comput. Biol. Bioinform..

[201] Lin S., Shi C., Chen J. (2022). GeneralizedDTA: Combining pre-training and multi-task learning to predict drug-target binding affinity for unknown drug discovery. BMC Bioinform..

[202] Yuan Q., Chen S., Wang Y., Zhao H., Yang Y. (2022). Alignment-free metal ion-binding site prediction from protein sequence through pretrained language model and multi-task learning. Brief. Bioinform..

[203] Mozhaeva V., Kudryavtsev D., Prokhorov K., Utkin Y., Gudkov S., Garnov S., Kasheverov I., Tsetlin V. (2022). Toxins’ classification through raman spectroscopy with principal component analysis. Spectrochim. Acta Part A Mol. Biomol. Spectrosc..

[204] Zou X., Ren L., Cai P., Zhang Y., Ding H., Deng K., Yu X., Lin H., Huang C. (2023). Accurately identifying hemagglutinin using sequence information and machine learning methods. Front. Med..

[205] Fujita T. (2011). In memoriam professor corwin hansch: Birth pangs of QSAR before 1961. J. Comput. Aided Mol. Des..

[206] Veerasamy R., Rajak H., Jain A., Sivadasan S., Varghese C., Agrawal R. (2011). Validation of QSAR models-strategies and importance. Int. J. Drug Des. Discov..

[207] Tropsha A. (2010). Best practices for QSAR model development, validation, and exploitation. Mol. Inform..

[208] He S., Ye X., Sakurai T. (2024). CLOP-hERG: The Contrastive Learning Optimized Pre-Trained Model for Representation Learning in Predicting Drug-Induced hERG Channel Blockers. Medinformatics.

[209] Xiang H., Zeng L., Hou L., Li K., Fu Z., Qiu Y., Nussinov R., Hu J., Rosen-Zvi M., Zeng X.J.N.C. (2024). A molecular video-derived foundation model for scientific drug discovery. Nat. Commun..

[210] Li T., Ren X., Luo X., Wang Z., Li Z., Luo X., Shen J., Li Y., Yuan D., Nussinov R.J.N.C. (2024). A foundation model identifies broad-spectrum antimicrobial peptides against drug-resistant bacterial infection. Nat. Commun..

